# *Acinetobacter* phages use distinct strategies to breach the capsule barrier

**DOI:** 10.1371/journal.ppat.1013536

**Published:** 2025-09-29

**Authors:** Alexis J. McCalla, Forrest C. Walker, Fabiana Bisaro, Miguel Rodriguez-Anavitate, Anna Johannesman, Gisela Di Venanzio, Mario F. Feldman, Michele LeRoux

**Affiliations:** Department of Molecular Microbiology, Washington University in Saint Louis School of Medicine, Saint Louis, Missouri, United States of America; University of Georgia, UNITED STATES OF AMERICA

## Abstract

*Acinetobacter baumannii* is an opportunistic pathogen that is a growing threat in hospital settings due to its alarmingly high rates of antibiotic resistance. Alternative therapies are urgently needed to manage the growing burden of untreatable *A. baumannii* infections. Phage therapy is a promising avenue that has already seen some success in isolated compassionate-use cases, including the famous “Patterson case”. Bacterial surface structures are the first determinants of susceptibility to phage infection and play a major role in phage host range. Most *A. baumannii* strains produce a protective capsule that is highly diverse both in structure and composition, and provides the first immunity barrier against phages. Here, we perform a detailed molecular characterization of three recently isolated, distinct *A. baumannii* phages, StAb1, StAb2, and StAb3, that breach the capsule via different mechanisms. Like many previously described *A. baumannii* phages, a specific capsule type is necessary for StAb1 infection. We found that StAb2 and its relatives adsorb to either a specific capsule type or the conserved outer membrane protein CarO, a porin normally occluded by the capsule. Thus, this phage has a narrow host range amongst capsulated strains, but can broadly infect *A. baumannii* strains lacking capsule. We also show that StAb3 requires a conserved and uncharacterized glycan that we have termed phage glycan receptor (PGR) that enables StAb3 to infect a broad range of *A. baumannii* strains irrespective of whether capsule is present. We demonstrate how rationally combining phages with distinct capsule interactions reduces the rapid emergence of phage escape mutants, with potential applications for more effective phage therapy.

## Author summary

*Acinetobacter baumannii* causes life-threatening and often antibiotic-resistant infections thereby posing a global threat. Accordingly, there is an urgent need for alternative treatments such as phage therapy, the use of bacterial viruses to treat infections. However, each Acinetobacter phage can typically only infect a few *A. baumannii* strains. This is largely due to a protective layer, called capsule, that surrounds the cell but whose composition varies widely between strains. In this study, we isolated and characterized three *Acinetobacter* phages (StAb1, StAb2, and StAb3). Many *Acinetobacter* phages, including StAb1 and StAb2, bind to and degrade a specific capsule type, infecting a limited number of strains. When infected, bacteria often quickly stop producing the capsule, thereby becoming phage resistant. However, StAb2 also infects strains without a capsule by attaching to a normally hidden protein on the cell surface. We show that by combining StAb1 and StAb2, or similar phages, *A. baumannii* is less likely to become resistant. Finally, we isolated StAb3 which infects a majority of *A. baumannii* strains by binding a previously unknown cell surface polysaccharide called PGR. By understanding how phages bind to and infect bacteria, we can design more effective phage therapy and uncover fundamental biology about this pathogen.

## Introduction

The opportunistic pathogen *Acinetobacter baumannii* causes severe, frequently untreatable nosocomial infections that are increasingly challenging to manage due to rising levels of multi-drug resistance (MDR) [1]. Accordingly, both the World Health Organization and the United States Centers for Disease Control and Prevention have determined that developing new treatments for MDR *A. baumannii* is an urgent priority [2,3]. The use of bacteriophages (phages) to treat bacterial infections has reemerged as a promising alternative approach for combating MDR pathogens [4–6]. However, the phages that infect *A. baumannii* have a notoriously narrow host range, typically infecting only a small handful of strains, thus limiting their utility in phage therapy [7–9]. Furthermore, even when patients are treated with phage cocktails to which the bacteria is susceptible, resistance often develops rapidly [6,10].

The most important factor that drives host range is the compatibility between the phage receptor binding protein (RBP) and the bacterial receptor, typically a protein or molecule exposed on the cell surface such as lipopolysaccharide or type IV pili [11,12]. In some cases, the bacterial capsule can serve a protective role, blocking access to receptors and providing phage resistance in organisms such as *Escherichia coli* and *Staphylococcus aureus* [13–15]. However, some bacteriophages are able to use capsule as a receptor, especially in species that are typically encapsulated such as *Klebsiella pneumoniae* [16]. Most *A. baumannii* phages characterized to date use the bacterial capsule as their receptor, with surprisingly few other receptors reported [4]. The *A. baumannii* capsule is a crucial virulence factor that protects the bacteria from various threats including killing by the eukaryotic host immune system as well as from small molecules like antibiotics [17–23]. The bacterial capsule is composed of capsular exopolysaccharides that come in a staggering number of variations in sugars and specific linkages, with over two hundred unique capsule loci producing over seventy distinct capsule structures in this organism [24]. Individual phages often carry a capsule-specific depolymerase associated with their tail spike protein that allows them to bypass this physical barrier [25]. Because of this narrow specificity, identifying phages for any given *A. baumannii* strain is challenging and bacteria quickly become resistant to capsule-dependent phages by modifying or losing their capsule both *in vitro* and *in vivo* [10,20,26,27]. A deeper understanding of the receptor usage of phages commonly used in phage therapy is crucial to circumventing bacterial resistance and rationally designing phage cocktails [28–30]. Despite the relatively low diversity of reported *Acinetobacter* phages, surprisingly little is understood about how these phages adsorb to their host.

In this work we report the isolation of three lytic phages, StAb1, StAb2, and StAb3, that represent the three major morphological types of double-stranded DNA phages (podo-, myo-, and siphoviruses). StAb1 and StAb2 represent two of the most commonly isolated groups of *Acinetobacter* phages, Friunaviruses and Twarogviruses, and are closely related to phages previously used in phage therapy [10,31]. We find that StAb1 and StAb2 are both highly specific for a particular capsule type, but intriguingly, StAb2 is also able to infect any *A. baumannii* strain tested lacking a capsule. In contrast, StAb3 is highly unusual in its ability to infect most *A. baumannii* strains irrespective of capsule type or capsule presence. Here, by leveraging escape mutant analyses, we identify the receptors for each of these phages, revealing different strategies employed by *Acinetobacter* phages to overcome the capsule barrier. We find that rationally combining phages based on their distinct capsule interaction strategies can reduce development of bacterial resistance, demonstrating potentially therapeutically useful insights into phage cocktail selection.

## Results

### Isolation and classification of three *A. baumannii* phages

**Fig 1:**
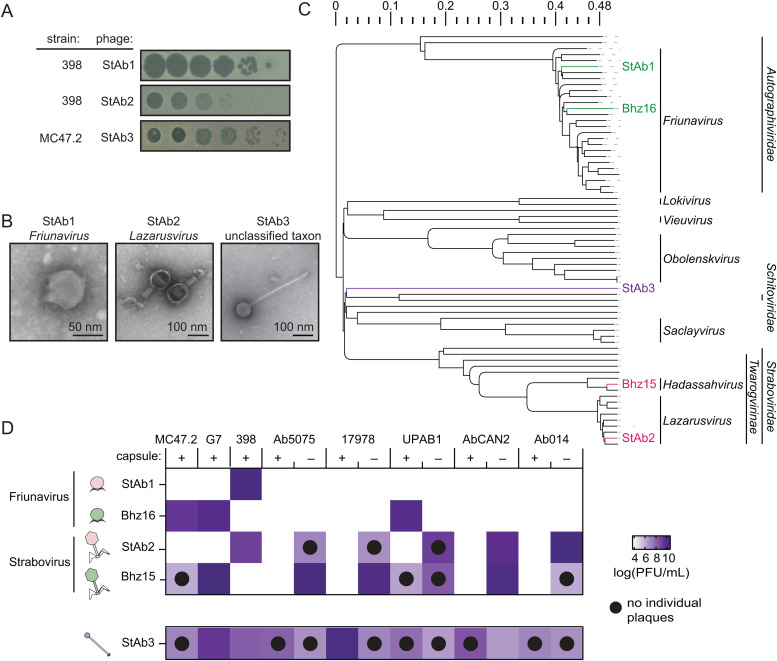
Isolation of three representative *A. baumannii* phages. A) Plaque assays of each phage on the indicated isolation host. B) TEM images of StAb1, StAb2, and StAb3. C) Phylogenetic tree of the phages used in this paper and the 65 *Acinetobacter*-infecting phages with complete genomes available in RefSeq, with phages used in this paper labeled. Scale indicates genomic distance, as defined previously [35]. D) Host range analysis of StAb1, Bhz16, StAb2, Bhz15, and StAb3 on a panel of *A. baumannii* strains with and without capsule with colors indicating the average log(PFU/mL) of three independent replicates. Phages were titered using double overlay plaque assays under optimal conditions for each phage, as described in Materials and Methods. Black dots indicate combinations where phages did not form individual plaques in at least 2/3 replicates.

We isolated three *A. baumannii* phages from wastewater using two different *A. baumannii* clinical isolate hosts: StAb1 and StAb2 were isolated on 398 and StAb3 was isolated on MC47.2. All phages form clear plaques and exhibit lytic activity against their isolation host strain (Fig 1A). Transmission electron microscopy (TEM) reveals that these are all tailed phages: StAb1 is a podophage with a head measuring 63 (±4.9) nm, StAb2 is a myophage with a hexagonal head of 78 (±4.6) nm width and 103 (±5.9) nm height, and StAb3 is a siphophage with a head about 75 (±2.4) nm in diameter, but with a notably long, non-contractile tail (336 ± 8.4 nm) (Fig 1B). We next sequenced the phage genomes and found that StAb1 has a genome of approximately 42 kb and is in the *Autographiviridae* family, genus *Friunavirus* (Figs 1C and S1A). StAb2 has a genome of approximately 165 kb and is a member of the *Straboviridae* family, which also contains the model *E. coli* T4 phage; within this family it belongs to the *Acinetobacter* specific *Twarogvirinae* subfamily (Figs 1C and S1B). Within this subfamily, StAb2 is a strain of *Lazarusvirus fhyacithree*, exhibiting >90% identity and substantial synteny to other phages of the *Lazarusvirus* genus, including a group of phages used in the Patterson phage therapy case (AC4, Navy1, Navy4, and Navy97) as well as two recently reported phages, DLP1 and DLP2, that were isolated on acapsular strains of *A. baumannii* [31]. StAb3 has a genome of approximately 79 kb and is a member of an unclassified taxon with similarity to three other taxonomically unclassified *Acinetobacter* phages described as having a broad host range, with 96.2% average nucleotide identity (ANI) to EAb13, 77.1% ANI to Mystique, and 76.8% ANI to vB_AbaS_TCUP2199, placing all of these phages within an unclassified and mostly unexplored shared genus [32–34] (Figs 1C and S1C–D).

We next determined the host range of these three phages across a panel of contemporary clinical isolates of *A. baumannii.* We observed that both StAb1 and StAb2 form plaques only on their isolation host, with no visible clearance or lysis visible on any other wild-type strains (Fig 1D). This narrow host range is similar to most known *Acinetobacter* phages, which typically use the highly variable capsule as the receptor, and we hypothesized that the host range of StAb1 and StAb2 is likewise dependent on capsule type. In contrast, StAb3, while not able to form visible individual plaques on every strain, is able to form zones of clearance at high titers for each of the tested clinical isolates (Fig 1D).

In the course of our host range experiments, we noted StAb2 could occasionally form plaques on one of the clinical isolates in our collection, Ab5075. Upon further investigation, we discovered that one of our glycerol stocks contained an Ab5075 variant that had spontaneously lost capsule, and that this capsule deficient strain is susceptible to StAb2 infection, while its wild-type counterpart is fully resistant. This surprising observation suggests that, in the absence of capsule, StAb2 may have a broader host range and that capsule is not a requirement for infection of StAb2, unlike many other reported *Acinetobacter* phages. We previously showed that a mutant in the initiating glycosyltransferase, PglC, in *A. baumannii* strain 17978 was unable to produce capsule and that this could be complemented by supplying *pglC* in a plasmid [36]. We confirmed these phenotypes by employing a previously validated density gradient assay [37–39] (S2A-B Fig). Bacteria with more capsule have a lower density, while bacteria with decreased or absent capsule have a higher density and thus travel further down the density gradient upon centrifugation [37–39]. Consistent with StAb2 infecting acapsular strains more broadly, we found that the *pglC* mutant is sensitive to StAb2, while resistance was restored in the complemented strain (S2C Fig). We assembled a panel of clinical isolates that had lost their capsule either through targeted deletions or spontaneous mutations and validated the loss of capsule with both sequencing and the density gradient assay (S2D Fig and S1 Table). We found that, consistent with most other reported *A. baumannii*-infecting phages, StAb1 retained its narrow host range, only forming plaques on its isolation strain, 398 (Fig 1D). However, StAb2 was able to infect capsule-deficient variants of five distinct clinical isolates of *A. baumannii* (Fig 1D), demonstrating that StAb2 has a surprisingly broad host range in the absence of capsule, implying that capsule functions as a barrier to infection by this phage. Finally, both the wild-type strains and capsule mutants are susceptible to StAb3, though in most cases the capsule mutants do show reduced sensitivity compared to the wild-type strains (Fig 1D). Together, these results demonstrate that phylogenetically diverse *Acinetobacter* phages exhibit key differences in their interactions with capsule, which can impede or promote replication of distinct phages.

### *Acinetobacter* phage StAb1 requires capsule for infection

**Fig 2:**
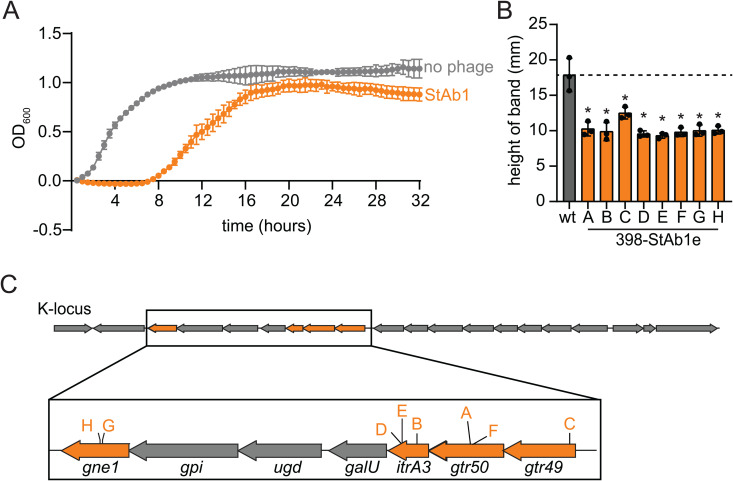
*A. baumannii* escapes StAb1 by losing its capsule. A) Bacterial growth of 398 monitored via optical density at 600 nm (OD_600_) with and without StAb1 phage at an MOI of 0.01. The average of three technical replicates is presented, error bars representing standard deviation (sd). B) StAb1 phage escape mutant capsule formation assessed via silica-based density gradient experiments and compared with the wild-type (wt) parental strain 398 (gray). Three independent replicates and their average is presented with error bars representing sd. Statistical significance was determined by an ANOVA, and each mutant was compared to the wild-type with Dunnett’s multiple comparisons test, * P < 0.05. C) 398 capsule biosynthesis operon (K-locus) indicating the location of SNPs for each 398-StAb1 escape mutant corresponding to strains in (B).

To systematically determine the key bacterial factors that contribute to phage host range, we first set out to analyze bacterial clones that had acquired resistance to StAb1. For StAb1 planktonic infection, we typically observed an initial period during which no bacterial growth is detected, followed by an increase in optical density, suggesting the presence of escape mutants (Fig 2A). Clones of the surviving bacteria were isolated and durable phage resistance was confirmed (i.e., no plaques observed in double overlay assays).

We assessed capsule production in these escape mutants using the density gradient assay and determined that all StAb1 escape mutants likely lost or produce dramatically less capsule (Fig 2B). Consistent with these results, 398-StAb1 escape mutants all have mutations in genes found in the K-locus, the locus encoding most genes required for capsule synthesis (Fig 2C and S2 Table) [24]. To extend these findings to the larger *Friunavirus* genus, we acquired a closely related phage, Bhz16, and found that while it has specificity for a different capsule type, the pattern is largely the same [40]. Bhz16 can form plaques only on three strains predicted to have capsule type K9, UPAB1, MC47.2, and G7, but is unable to replicate on an acapsular derivative of UPAB1 or any other acapsular strain (Fig 1D and S3 Table) [24,41]. As with StAb1, G7-Bhz16 escape mutants have reductions in capsule and mutations in the K-locus (S2E Fig and S2 Table). This requirement for capsule likely drives the very limited host range of *Friunavirus* phages, with only capsule types complementary to each phage able to support infection.

### Bacteria can become resistant to StAb2 by increasing or altering capsule

**Fig 3:**
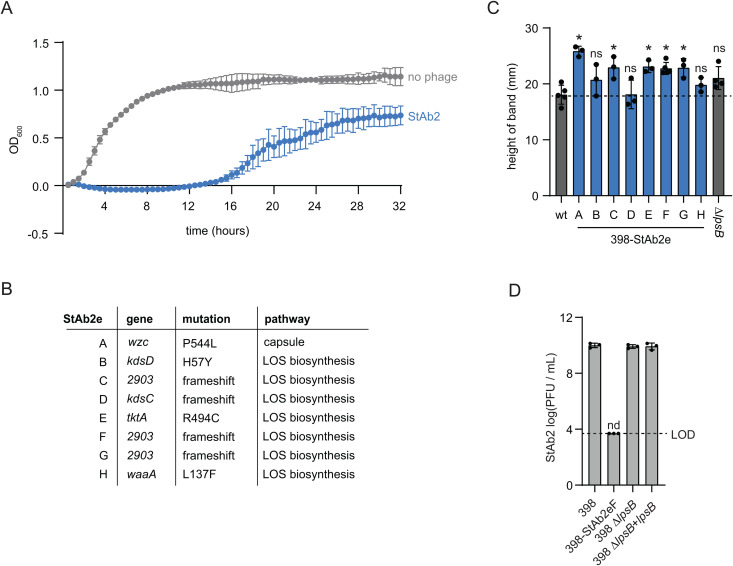
*A. baumannii* evades StAb2 by altering or increasing capsule. A) Bacterial growth of 398 monitored via OD_600_ rebounds after StAb2 phage addition at an MOI of 0.01. The average of three technical replicates is presented with error bars representing sd. B) Summary of mutations identified in 398-StAb2 escape mutants. C) StAb2 phage escape mutant capsule formation assessed via silica-based density gradient experiments and compared with the wild-type (wt) parental strain 398 (gray). Statistical significance was determined by an ANOVA, and each mutant was compared to the wild-type with Dunnett’s multiple comparisons test, * P < 0.05, ns P > 0.05. D) Quantification of plaque assays with StAb2 on the indicated strains. Limit of detection (LOD) = 3.7. nd: not detected. C-D) Independent replicates and their average is presented with error bars representing sd.

We also obtained isolates of *A. baumannii* 398 that were resistant to StAb2 (Fig 3A). Sequencing revealed a more diverse suite of mutations in StAb2 resistant strains than those found for StAb1. One escape mutant, 398-StAb2eA, mapped to the K-locus (Fig 3B and S2 Table). We noted that when grown on an agar plate, this strain is more mucoid than the wild type, and its migration in the density assays suggests increased capsule production (Fig 3C). This strain has a point mutation in *wzc*, which encodes a tyrosine kinase that plays a key role in regulation of capsular polysaccharide synthesis and transport across the membrane [42]. Specific point mutations in *wzc* have been previously shown to result in overproduction of capsular polysaccharides [23,43]. All other StAb2 escape mutants have single nucleotide polymorphisms (SNPs) in genes associated with lipooligosaccharide (LOS) synthesis. These include mutations in A1S_2903, a gene involved in an early step of LOS synthesis [44]. We therefore hypothesized that LOS was essential for StAb2 infection, potentially serving as a receptor. However, contrary to this hypothesis, we found no change in susceptibility for a Δ*lpsB* mutant to StAb2 (Fig 3D). It was previously shown that ΔA1S_2903 and ∆*lpsB* synthesize the same truncated LOS, indicating that changes in LOS do not drive StAb2 resistance in these strains and that LOS is not the receptor for StAb2 [44]. Density gradient assays of the StAb2 escape mutants demonstrate that *wzc, tktA*, and A1S_2903 mutants (A, C, F, G) have increased capsule relative to wild type, while the *kdsC*, *kdsD*, and *waaA* escape mutants and Δ*lpsB* mutant have no statistically significant changes in capsule thickness (Fig 3C). This, together with the reduced sensitivity of these strains to StAb2, suggests potential unexplored crosstalk between LOS and capsule synthesis in *A. baumannii* that should be investigated further in the future. In conclusion, in contrast to StAb1, StAb2 escape mutants are not deficient in capsule synthesis, and instead likely acquire resistance through increased or altered capsule. Importantly, some form of capsule, possibly altered, is maintained, consistent with our observations that acapsular strains are susceptible to StAb2 infection. Together, these data demonstrate that the capsule provides a critical first line of defense against StAb2.

### *Twarogvirinae* phages use an outer membrane porin, CarO, as an alternate receptor

**Fig 4:**
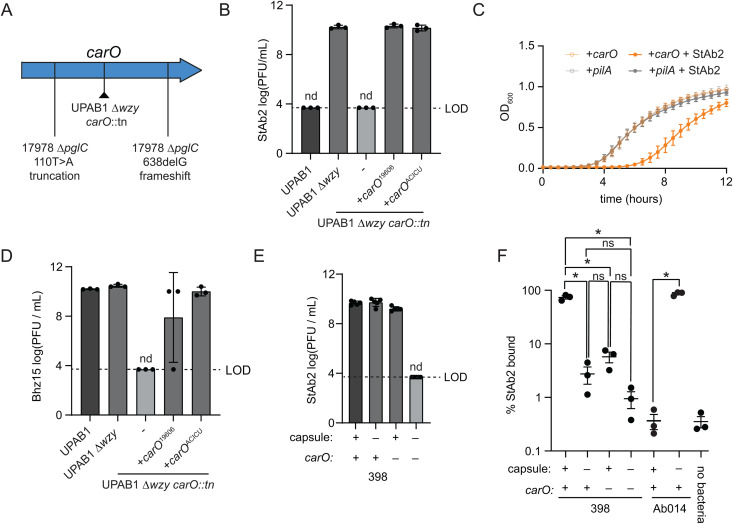
CarO is an alternate receptor for StAb2 in the absence of capsule. A) Mutations identified in CarO for StAb2 resistant strains: 17978 ∆*pglC*-StAb2eA-H (A, D, G, and H with 110T > A and B, C, E, and F with 638delG) and UPAB1∆*wzy carO*::tn, as indicated in the schematic. See S2 Table for details about escape mutants. B) Quantification of plaque assays with StAb2 on the indicated UPAB1 strains and mutants complemented with *carO* from the indicated strain. C) Growth of *E. coli* expressing either CarO or PilA (control) with or without addition of StAb2 at MOI = 1000. The average of three independent replicates each with three technical replicates is presented with error bars representing sd. D) Quantification of plaque assays with Bhz15 on the indicated UPAB1 strains ectopically expressing different *carO* alleles, as in (B). E) Quantification of plaque assays with StAb2 on the indicated strains: 398 (capsule + , CarO+), 398-StAb1eA (capsule-, CarO+), 398 ∆*carO* (capsule + , CarO-), or 398-StAb1eA ∆*carO* (capsule-, CarO-). Five independent replicates and their average is presented with error bars representing sd. LOD = 3.7. nd = not detected. F) Adsorption of StAb2 after 10 min of incubation with the 398 strains from (E), as well as Ab014 (capsule +) and Ab014-cm (capsule -). Statistical significance was determined by an ANOVA, and each strain was compared with Šidák’s multiple comparisons test, * P < 0.05, ns P > 0.05. B, D, F) LOD = 3.7. nd = not detected. Three independent replicates and their average is presented with error bars representing sd.

To identify the non-capsular receptor for StAb2 we employed a transposon mutagenesis-based screening approach. We generated a high-density transposon mutant library of one of the StAb2-sensitive, capsule-deficient strains, UPAB1 ∆*wzy,* challenged the library with StAb2, and isolated surviving mutants. Phage resistance was verified by plaque assays, and we then identified the transposon insertion sites by sequencing. Three resistant mutants contained transposon insertions in the gene encoding the outer membrane porin, CarO (Carbapenem-associated outer membrane protein), suggesting that CarO may be the receptor for this phage (Fig 4A). Additionally, we generated escape mutants to StAb2 in the acapsular 17978 ∆*pglC* strain and found that each clone has a SNP in the *carO* gene. Four of these candidates have a SNP at position 110 in the *carO* nucleotide sequence, resulting in a premature stop codon and truncation of the CarO protein, while the remaining four have a SNP at position 638 (-G), resulting in a frameshift mutation (Fig 4A and S2 Table). CarO is a conserved porin involved in nutrient uptake [45–47]. While the gene *carO* is interrupted or absent in some *A. baumannii* isolates, it is present in most *A. baumannii* genomes, explaining why StAb2 can infect any of the capsule-deficient strains tested [46,48,49]. Some studies suggest a role for CarO in carbapenem uptake, with loss or mutation of CarO leading to resistance to carbapenems like imipenem [45,49–51]; however, other work suggests CarO does not transport imipenem [47,52]. To determine if developing resistance to StAb2 by loss of CarO is likely to lead to resistance to imipenem, we measured changes in the imipenem minimum inhibitory concentration between parental and *∆carO* for four strain pairs, and found only minor differences (<2-fold), suggesting loss of CarO does not result in a significant increase in imipenem susceptibility in these strains(S4 Table).

We identified and cloned CarO proteins from two major CarO subtypes (S3 Fig), and found that both were able to complement the transposon insertion strain and restore phage replication when expressed ectopically (Fig 4B) [49]. Further validating that CarO serves as a receptor for StAb2, we also found that when CarO^19606^ is expressed in *E. coli*, StAb2 was able to inhibit growth in planktonic infections (Fig 4C). We did not observe this StAb2-dependent growth defect in *E. coli* expressing PilA, which was employed as a control. Together, these experiments demonstrate that for an acapsular *A. baumannii*, CarO is necessary for StAb2 infection, and that expression of CarO is sufficient for phage infection in even a distantly related bacterial species.

We hypothesized that *Twarogvirinae* carry a conserved CarO-binding protein in addition to a unique capsule-binding (and/or degrading) protein specific for the capsule type of its host. Supporting this idea, *carO* was identified in an escape mutant analysis of a related *Lazarusvirus*, Navy-1, following *in vitro* selection, though the authors did not consider a role for *carO* as a potential phage receptor [10]. These authors also noted mutations in the K-locus in resistant bacteria arising both *in vivo* and *in vitro,* though whether these strains had lost capsule was never tested. Further, CarO was also identified as the receptor for the *Lazarusvirus* DLP2, a phage isolated on an acapsular strain [31]. To test whether the capsule/CarO dual receptor usage is similar for another *Twarogvirinae* phage of a different genus, we obtained a related phage, Bhz15, (subfamily *Twarogvirinae*, genus *Hadassahvirus*) [40]. Among encapsulated strains, Bhz15 has a narrow host range, like Bhz16 infecting only strains with predicted capsule type K9 (Fig 1D and S3 Table). Much like StAb2, Bhz15 can also infect all acapsular *A. baumannii* strains (Fig 1D). Bhz15 readily infects the capsule mutant UPAB1 ∆*wzy*, but was unable to infect UPAB1 ∆*wzy carO*::tn, a mutant resistant to StAb2 with a transposon interrupting CarO, which can be complemented via ectopic CarO expression (Fig 4D). This demonstrates that Bhz15, like StAb2, is prevented from accessing CarO by most capsule types, and in the absence of capsule, can use CarO as a receptor, suggesting that this receptor usage pattern is a feature of the *Twarogvirinae*.

### StAb2 can use either CarO or cognate capsule for cell entry

While CarO is clearly essential for infection in non-isolation hosts and sufficient for infection of entirely distinct bacteria, StAb2 and Bhz15 can each replicate on their capsule-proficient isolation strains, 398 and G7, respectively. In these strains, CarO is likely cloaked by the presence of capsule, implying these phages utilize the capsule or another external structure as a primary receptor. We wondered if CarO was required for infection of 398 by StAb2, or if the capsule could serve as the sole receptor. To test this, we generated a *carO* deletion in both the wild-type 398 and one of the capsule-deficient 398 StAb1 escape mutants (398-StAb1eA) and tested StAb2 infectivity and adsorption with these strains. StAb2 has a similar efficiency of plating (EOP) on 398-StAb1eA, which lacks capsule, compared with ∆*carO* and the wild-type 398 (Fig 4E). Similarly, in planktonic infection, StAb2 was able to inhibit the growth of the capsule deficient and ∆*carO* strain (S4 Fig). We noted that the ∆*carO* mutant develops escape mutants more consistently than the wild-type 398 (S4 Fig). The strain lacking both capsule and CarO, 398-StAb1eA ∆*carO*, is resistant to StAb2 in plaque assays and in planktonic culture, demonstrating that StAb2 requires either CarO or capsule for infection (Figs 4E and S4 Fig). Indeed, direct measurement of adsorption revealed that about 80% of the bacteriophages adsorb to wild-type 398 within 10 minutes, about 10% can adsorb to 398 bearing only intact capsule or CarO, while phage adsorption to a strain lacking both receptors is approaching background levels (Fig 4F). Together, this demonstrates that StAb2 can use either CarO or capsule in infecting 398, though adsorption is considerably improved by having both.

To assess if the inability of StAb2 to infect most other capsulated *A. baumannii* strains is due to an inability to adsorb or to penetrate the cell wall, we assessed the ability of StAb2 to adsorb to wild-type clinical isolate Ab014 and its capsule mutant counterpart. Wild-type Ab014 produces capsule and is K-locus type 120, distinct from that of the StAb2-susceptible encapsulated 398 with K-locus type 24 (S3 Table). We found that StAb2 is entirely unable to adsorb to the wild type, but after 10 minutes, close to 100% of phage particles have adsorbed to the Ab014 capsule mutant (Fig 4F). This demonstrates that while StAb2 can adsorb to its cognate capsule type found in 398, it is unable to adsorb to a strain with a non-cognate capsule type, Ab014. Further, this demonstrates that the *A. baumannii* capsule does indeed prevent StAb2 from accessing its receptor, CarO. StAb2 adsorption to acapsular Ab014 is also notably stronger than to acapsular 398, though the weaker adsorption to acapsular 398 does not meaningfully impact phage replication (Fig 4E).

While Bhz15 and StAb2 can both infect acapsular *A. baumannii* strains using CarO as a receptor, their host range amongst capsular *A. baumannii* strains is distinct. The ability of phages to penetrate extracellular polysaccharides such as capsule or biofilms is often dependent on the presence of phage-encoded depolymerases [25,53,54]. Analysis of the proteins encoded by StAb2 using two machine learning tools that predict phage depolymerases both identified CDS_0239, annotated as a tail fiber protein, as the most likely depolymerase (S5A Fig) [55,56]. Characterized depolymerases in *Acinetobacter*-infecting Friunaviruses are similarly tail spike proteins and are encoded directly before lysins, as is CDS_0239 [25]. A closer examination of the tail fiber genes revealed that, while most genes are very similar within the *Lazarusvirus* and *Hadassahvirus* genera, homologs of CDS_0239 exhibit substantial variation that is inconsistent with broader phylogeny, reminiscent of the hypervariable regions found within the *A. baumannii* K-locus (S5A–C Fig) [24,57]. This hypervariability further supports the hypothesis that CDS_0239 is the likely depolymerase, as it must rapidly evolve to adapt to different capsules. A multiple sequence alignment of this protein with its homologs from other annotated Lazarusviruses and Hadassahviruses revealed considerable mosaicism, with the last ~250 amino acids exhibiting the highest variability of this protein (S5D Fig). This C-terminal domain varies substantially between closely related phages and does not track with phylogeny, suggesting it is under strong positive selection, similar to *Friunavirus* tail spike proteins, wherein the N-terminal portion is highly conserved and the divergent C-terminal domains have been proposed to determine specificity for capsular polysaccharides (S5D Fig) [25]. The second variable region of this protein (amino acids 69–976) contains the previously identified pyocin knob domains which are variable between *Lazarusvirus* and *Hadassahvirus* strains and are proposed to be involved in receptor interactions [58].

Based on these analyses and our experimental observations, *Twarogvirinae* phages StAb2 and Bhz15 attach to and likely degrade the capsule of susceptible hosts using the highly variable long tail fiber protein, and then use a second, conserved receptor-binding protein to bind CarO. Thus, the *A. baumannii* capsule blocks access to CarO from the vast majority of Twarogvirinae phages except for the small subset with a depolymerase capable of degrading its particular capsule type (e.g., StAb2 for 398 capsule; Bhz15 for G7 capsule). However, they can infect acapsular *A. baumannii* strains as the CarO protein is exposed.

### StAb3 requires an uncharacterized cell surface polysaccharide for infection

**Fig 5:**
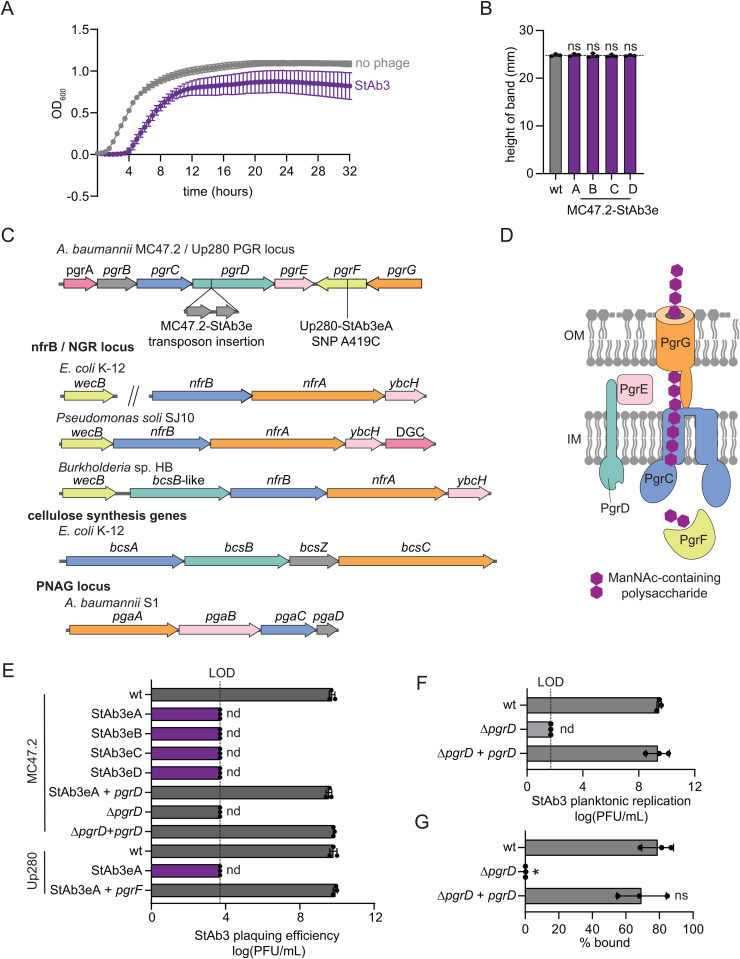
Escape mutants to StAb3 arise in a predicated EPS biosynthesis and secretion pathway. A) Bacterial growth of MC47.2 monitored via OD_600_ rebounds after StAb3 phage addition at an MOI of 1, suggesting phage-insensitive bacteria arise rapidly. The average of three biological replicates each with six technical replicates is presented with error bars representing sd. B) StAb3 phage escape mutant capsule formation assessed via silica-based density gradient experiments and compared with the wild-type (wt) parental strain MC47.2 (gray). C) Locus containing a transposon insertion in MC47.2 and Up280 escape mutants to StAb3 infection. Shared predicted function and homology to related EPS export pathways is indicated by color, see S6 Fig and S5 Table. D) Model of predicted exopolysaccharide secretion system based upon the structural homology and localization of proteins in related EPS secretion systems. Predicted protein localization in the cytoplasm, inner membrane (IM) or outer membrane (OM) is shown. E) Quantification of plaque assays with StAb3 on the indicated strains. LOD = 3.7. F) Final StAb3 titer after planktonic replication with wild-type MC47.2 (wt) and *pgrD* mutant and complement strains. LOD = 1.7. G) Adsorption of StAb3 after 10 min of incubation with the MC47.2 strains from (D). B,E-G) Three independent replicates and their average is presented with error bars representing sd. For B and G, statistical significance was determined by an ANOVA, and each strain was compared to the wt with Dunnet’s multiple comparisons test, * P < 0.05, ns P > 0.05. For E and F nd = not detected.

In contrast to StAb1 and StAb2, StAb3 is able to infect a wide range of *A. baumannii* strains, including both capsular and acapsular strains (Fig 1D). We also tested StAb3 replication in planktonic culture and found that it inhibits growth, though does not lead to complete lysis of the culture. As with StAb1 and StAb2, we also see bacterial growth resume after a period of cessation, presumably due to bacterial resistance arising (Fig 5A). It seemed unlikely that a depolymerase could target multiple, diverse capsule types, as they are often composed of entirely unique polysaccharides, and further, StAb3 could infect entirely acapsular strains as well. To investigate how StAb3 is able to infect diverse capsule types, we obtained MC47.2 escape mutants from StAb3. None of the strains recovered in the planktonic growth experiments proved stably resistant, so escape mutants were instead isolated from top agar. All of these escape mutants were comparable to the wild type in the density gradient assay, suggesting that the capsule thickness is unchanged (Fig 5B). All isolates have two IS4 family transposase genes, also found in another section of the wild-type chromosome, inserted in the gene ABUS04_RS10855 (Fig 5C and S2 Table). This gene product is homologous to BcsB, a protein that in other bacterial species is involved in the synthesis of cellulose, suggesting StAb3 may be adsorbing to a non-capsular glycan, which we have termed phage glycan receptor (PGR) [59–62] (Figs 5C-D and S6 and S5 Table). To investigate this idea, we analyzed the locus in which the BcsB homolog is encoded, which we now call the PGR locus. The locus contains homologs to genes involved in the synthesis of other exopolysaccharides, including cellulose and Poly-N-acetylglucosamine (PNAG) [62] (Figs 5C-D and S6 and S5 Table). PgrC is homologous to NfrB, BcsA, and PgaC, all involved in exopolysaccharide synthesis and transport across the inner membrane; PgrG is predicted to be a putative outer membrane porin to facilitate polysaccharide transport across the outer membrane; and PgrE shares structural homology to the C-terminus of PgaB, involved in PNAG binding during its synthesis [62,63] (Figs 5C-D and S6 and S5 Table). A subset of the related exopolysaccharide loci are regulated by cyclic-di-GMP; we find that PgrA has structural homology to diguanylate cyclases but does not carry the catalytic GGDEF motif, and thus its role is unclear (S6 Fig and S5 Table). PgrF is homologous to WecB, a UDP-N-acetylglucosamine-2-epimerase, which synthesizes UDP-N-acetylmannosamine (ManNAc) (Figs 5C-D and S6 and S5 Table). A StAb3 escape mutant we isolated from a different parental *A. baumannii* strain, Up280, carries a single SNP within this *wecB* homolog (Fig 5C and S2 Table) [63,64]. Previous studies identified a similar operon in *E. coli* as producing a sugar essential for the bacteriophage N4 to adsorb to and infect *E. coli* [65,66]. The authors proposed that these genes encode an exopolysaccharide transport system producing a ManNAc-based polysaccharide that serves as the receptor for N4 [65,66]. Similarly, we propose that the PGR locus produces a still uncharacterized ManNAc-containing exopolysaccharide that serves as a receptor for StAb3 (Fig 5C-D). This polysaccharide likely protrudes beyond the capsule, providing StAb3 with a point of attachment and enabling infection of *A. baumannii* independent of capsule.

To further test the hypothesis that StAb3 binds to PGR, we performed an additional genetic analysis. Both the MC47.2 mutants with insertions in *pgrD* and the Up280 mutant with a SNP in *pgrF* are resistant to StAb3 based upon a plaque assay, with susceptibility restored by complementation (Fig 5C and 5E). Further, a clean deletion of *pgrD* in MC47.2 results in similar resistance to StAb3 in plaque assays and in planktonic growth, with susceptibility restored upon complementation (Fig 5E-F). Further, StAb3 readily adsorbs to MC47.2, with over 75% of phage bound within 10 minutes, but is completely unable to adsorb to the MC47.2 ∆*pgrD* strain (Fig 5G). We note that infection is in some cases diminished when capsule is removed, as reported previously for Mystique (Fig 1D); one potential explanation for this phenotype is that the capsule may help orient the phage and/or sugar, and when it is absent, infection proceeds with a reduced efficiency. Taken together, these data indicate that a previously unknown glycan, PGR, is the receptor to which StAb3 adsorbs.

### PGR is conserved among pathogenic Acinetobacter and is predictive of susceptibility to StAb3-like phages

**Fig 6:**
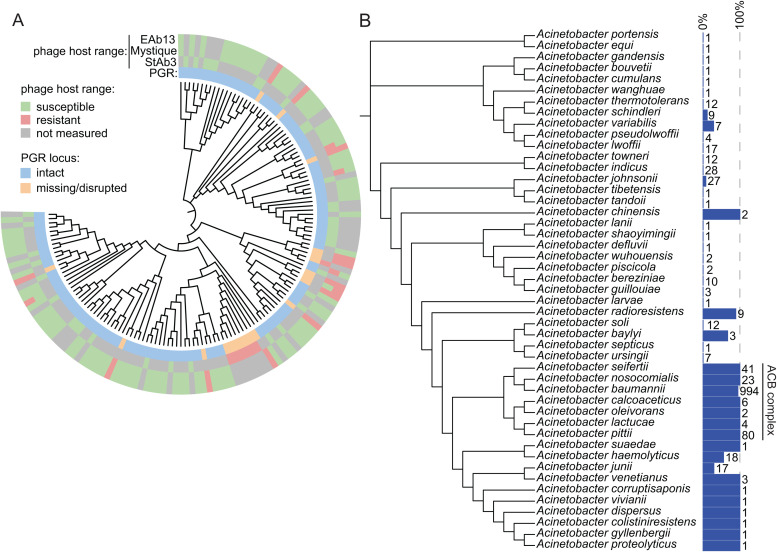
Escape mutants to StAb3 arise in a predicted EPS biosynthesis and secretion pathway. A) Phylogenetic tree of *A. baumannii* strains indicating presence of the PGR locus and susceptibility to StAb3, Mystique, and EAb13 moving from the inner ring to the outer ring. EAb13 and Mystique data are adapted from Margulieux et al. and Alseth et al. [32,33]. B) Phylogenetic tree of *Acinetobacter* species with a bar graph indicating percentage of strains within that species in NCBI that encode the PGR locus, with the number of genomes assessed listed to the right of the blue bars.

To further validate host range and phage dependence on PGR for StAb3 and related phages, we assessed susceptibility of 39 additional *A. baumannii* clinical isolates to StAb3, finding that StAb3 was able to infect 31 of these strains, and the 8 it was unable to infect had a missing or disrupted PGR locus, supporting a clear role for PGR in determining StAb3 host range across a large number of *A. baumannii* isolates (Fig 6A and S6 Table). Considering the unclear genetic underpinnings of the extremely broad host range of the related *Acinetobacter* phages EAb13 and Mystique, we examined whether variations within the PGR locus may also explain the widespread susceptibility to these phages, which were reported to infect 86% and 85%, respectively, of the MRSN *A. baumannii* isolates in plaque assays [32,33]. As reported previously for Mystique, there is no clear phylogenetic relationship determining susceptibility to Mystique or EAb13, though the authors propose a heritable trait is associated with susceptibility (Fig 6A). However, while 98.8% of the Mystique-susceptible and 100% of the EAb13-susceptible *A. baumannii* isolates from the MRSN collection bore a complete, intact PGR locus without any pseudogenes, complete loci could only be identified in 4/15 (26.7%) of Mystique-resistant and 2/14 (14.1%) of EAb13-resistant isolates (Fig 6A and S6 Table). The other phage-resistant isolates either lacked the locus, contained one or more pseudogenes, or could not be conclusively described due to being at the ends of contigs (S6 Table). The remaining related phage TCUP2199 was reported to similarly infect 91% of isolates, but we were unable to assess whether this interaction is also PGR-related due to the lack of genome sequences for tested strains. The small portion of strains which contain a PGR locus, but did not support infection, may be explained by the fact that this pathway is entirely uncharacterized and thus we have overlooked mutations that disrupt function, or these strains may carry defense systems active against these phages.

We further set out to understand how prevalent the PGR locus is among additional *A. baumannii* strains. A homology analysis of available *A. baumannii* genome sequences revealed that the PGR locus is nearly omnipresent within this species. A BLASTn-based search identified at least partial PGR loci in 958/994 (96.4%) of complete *A. baumannii* genome sequences. Manual inspection of PGR-negative genomes (3.6%) demonstrated that these strains often specifically lost the seven genes discussed above, as well as one directly neighboring gene, while maintaining surrounding gene content. Further clustering analyses of all PGR loci identified 20 distinct locus compositions, with 3.5% of PGR-positive strains carrying deletions of one or more genes and/or insertions into this region (S7 Fig). This subset of isolates often bear insertions of IS4 or other transposases within the PGR locus, similar to what we observed in our phage escape mutants. These mutations may have arisen during natural infection of these isolates by a StAb3-like phage, indicating a potential selective pressure mediated on *A. baumannii* cell surface structures by these poorly characterized phages. Similar to what we observed in the set of strains for which a StAb3-like phage has been assessed for infection, up to 93% of this wider diversity of *A. baumannii* strains appear to carry an intact locus. However, some of these loci that appear intact likely further carry pseudogene versions of one or more genes that may likewise disrupt function, as we have seen in some StAb3-like resistant strains (S6 Table).

The PGR locus is also present in other members of the *Acinetobacter* genus, but is notably enriched within the pathogenic *A. calcoaceticus-baumannii* (ACB) complex (Fig 6B). We thus investigated the ability of more distantly related, PGR-positive strains to support StAb3 infection. Strikingly, we found that, like Mystique, StAb3 can infect multiple non-*baumannii* strains that possess the PGR locus, indicating that the host range of this phage extends beyond a single narrow group of species (S8 Fig and S6 Table). These analyses together indicate that the PGR locus produces an unknown polysaccharide receptor that is utilized by multiple related *Acinetobacter* phages to infect a diversity of strains.

### Phages can be paired to counteract *A. baumannii* resistance

**Fig 7:**
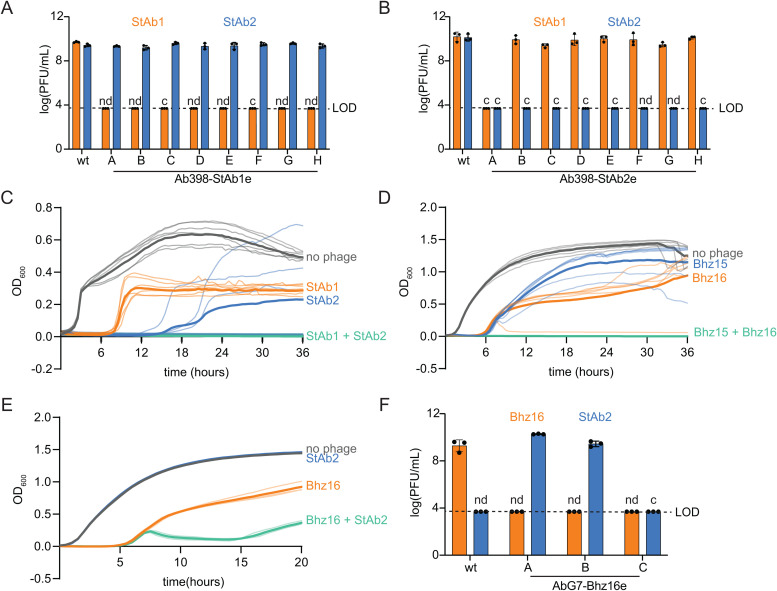
Combinations of phages prevent or delay bacterial resistance. A-B) Quantification of plaque assay with StAb1 and StAb2 on wild-type 398 and 398 StAb1 (A) and 398 StAb2 escape mutants **(B)**. LOD = 3.7. nd = not detected. c = areas of clearing, but no individual plaques, observed. C-E) Growth of 398 (C) or G7 (D-E) with the indicated individual phages or phages combined. Phages were added to an MOI of 1, either individually or combined. The average of six (C&D) or three (E) technical replicates is presented in bold lines with each replicate shown as a narrower line. See S9 Fig for two additional biological replicates and S10 for AUC comparisons. F) Replication of Bhz16 and StAb2 on either wild-type G7 or G7 Bhz16 escape mutants as in (A) and (B).

We have identified capsule-dependent (StAb1 and Bhz15) and capsule-restricted (StAb2 and Bhz16) *A. baumannii* phages. These findings suggest an intriguing possibility: capsule-restricted phages should be able to suppress the growth of capsule-deficient escape mutants that emerge rapidly when bacteria encounter capsule-dependent phages, such as StAb1, and extend the efficacy of these phages in combination. Multiple previous publications have demonstrated the benefit of combining capsule-dependent phages with capsule-restricted phages in phage cocktails [20,67,68]. Supporting this concept, escape mutants to StAb1 remain susceptible to StAb2, and most escape mutants from StAb2 remain susceptible to StAb1 (Fig 7A–B). Further, we found that when 398 (for StAb1 and StAb2) or G7 (for Bhz15 or Bhz16) is treated with a single phage, escape mutants arise within 20 hours and expand rapidly (Figs 7C-D, S9A-B and S10A-B). However, the combination of StAb1 and StAb2 on 398 or Bhz15 and Bhz16 on G7 fully suppress emergence of escape mutants throughout the entire course of our experiments, 36 hours (Figs 7C-D, S9A-B and S10 A-B).

One major hurdle with phage therapy is the need to either test many phages to identify one that can kill the bacteria causing the infection or even to isolate phages on that exact strain, due to their typically exquisite host-range specificity. However, our findings that Twarogviruses StAb2 and Bhz15 can infect a broad range of acapsular strains suggests that these phages could be used as a secondary phage in a cocktail against many *Acinetobacter* strains as long as a single capsule-dependent phage can be found that infects that strain, reducing labor required to isolate multiple strain-specific phages. We thus reasoned that StAb2 or Bhz15 should suppress escape mutants from arising in a non-native host strain when paired with a strain-specific, capsule-dependent phage that drives the emergence of acapsular escape mutants. To test this, we first combined Bhz15 with StAb1 to kill 398, which Bhz15 cannot typically infect, and found that the addition of Bhz15 was not able to prevent bacterial resistance from arising (S9C and S10C Figs). This is possibly due to a 398 host-specific factor such as a phage defense system that restricts Bhz15. However, we further combined StAb2 with Bhz16 on G7, which StAb2 cannot typically infect. While it did not fully prevent bacterial resistance from arising, it did delay and reduce escape mutants (Figs 7E, S9D and S10D). We also tested the resistant bacterial isolates following Bhz16 infection and found that two out of the three were susceptible to StAb2 (Fig 7F), suggesting that these phages could also function effectively if given sequentially rather than in combination. In summary, these data demonstrate that bacterial resistance can be eliminated or reduced by pairing a capsule-dependent phage with a *Strabovirus* that uses CarO as its receptor.

## Discussion

**Fig 8:**
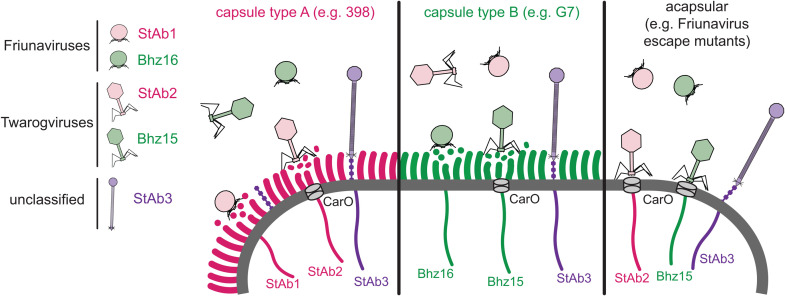
Model of bacteriophage-capsule interactions. Model for *Friunavirus* (StAb1-like), *Twarogvirinae* (StAb2-like), and StAb3-like phage interaction with *A. baumannii* capsule.

Capsule constitutes a formidable barrier that protects bacteria from diverse environmental stressors, including phages. In this work we describe three mechanisms by which diverse phages interact with or bypass this physical barrier (Fig 8). The selective pressure imposed by phage infection likely plays a role in driving the extensive diversification of the capsule. *A. baumannii* produce more than seventy types of capsule, which provides the first immunity barrier against phages [24]. Phages like StAb1 and StAb2 overcome this defense by attaching to and degrading the capsule to access the cell surface. While this seems to be necessary for StAb1 infection, StAb2 and other *Twarogvirinae* phages can also use CarO as a receptor, which is typically occluded by capsule. Both capsule-specific phages encode predicted depolymerases that are highly variable even within very close relatives [69]. The diversity of *A. baumannii* capsular structures is likely matched by specific and diverse depolymerases in these phages, which results in an effective phage attack with a narrow host range. A less well-characterized siphophage, StAb3, appears to employ an alternative strategy. Our experiments strongly suggest that this phage, which has a notably long tail, instead recognizes a yet to be characterized ManNAc-containing glycan, somehow being able to inject its DNA without depolymerizing the capsule.

Our study demonstrates the importance of phenotypically characterizing escape mutants, in addition to identifying genetic changes. Prior studies generally assumed that mutations in the K-locus are indicative of capsule loss; however, we find that one mutation located in the K-locus that led to StAb2 resistance has an increase in capsule production, rather than capsule loss, shown by our density-gradient assay. Furthermore, when we isolated 398 StAb2 escape mutants we found that most had SNPs in genes related to LOS synthesis, including A1S_2903. At first glance, this would suggest that StAb2 might use LOS as a receptor. However, a *lpsB* mutant, which in other strains has the same truncated LOS as a A1S_2903 mutant, remains susceptible to the phage, ruling out a role for LOS as the receptor [44]. Previous research demonstrated that while A1S_2903 and *lpsB* mutants have the same LOS structure, they are phenotypically distinct, with A1S_2903 mutants having a more pronounced growth defect, increased sensitivity to cell-wall targeting antibiotics, vancomycin and bacitracin, and increased propensity to form chains and clusters [44]. Many of the escape mutants we isolated, including those with SNPs in A1S_2903, migrate differently in the density gradient assay, suggesting increased or altered capsule. Thus, phenotypic characterization demonstrates mutations in the LOS biosynthesis pathway may affect capsule, revealing unexpected interactions between the LOS and capsule biosynthesis pathways. Our work shows that phages exert unique selective pressures that can lead to changes in bacterial physiology, revealed by escape mutant analyses, that expose knowledge gaps about basic bacterial processes, such as the unexpected cross-talks between LOS and capsule synthesis. More research is needed to understand how these pathways are interconnected and impact the assembly of the capsule.

CarO has been previously associated with resistance to *Twarogvirinae* phages. In a genomic analysis of the phages used for therapy in the Patterson case, an escape mutant with resistance to phage AB-Navy1 was isolated and has a mutation in *carO*; however, the authors proposed that CarO might be changing the cell wall and did not discuss the possibility that CarO might be acting as a receptor [10]. It is not clear why this mutation in CarO alone would be sufficient to abolish phage infection unless the capsule in this strain is not able to be used as a receptor and is not masking CarO to the same extent. Perhaps the strain produces less capsule *in vitro* due to the lower selective pressure for capsule, while *in vivo*, capsule production is higher and thus losing CarO alone would never be sufficient to lead to full resistance. Without a phenotypic analysis of capsule, it is impossible to distinguish between these possibilities. A loss of CarO has also been implicated in resistance to carbenicillin and thus the potential for StAb2-like phages to drive *A. baumannii* towards a more drug-resistant state should be taken into account when designing phage therapy cocktails. We did not detect a CarO-dependent change in imipenem resistance in our strains, thus the role of CarO in antibiotic resistance would need to be further investigated. Yet it was notable that CarO mutations were not identified *in vivo* in the Patterson case, consistent with reports that CarO may play a role in inhibiting host immune response and facilitating adherence to and invasion into cells [46,70–72]. In another report, CarO was identified as the receptor of the DLP2 phage, which is related to StAb2 [31]. In that work, the authors identified CarO through a transposon screening in a capsule deficient mutant [31]. However, the role of CarO in capsulated strains was not investigated. Since multiple Twarogviruses can interact with CarO, we speculate that the CarO binding protein is present in all of these phages and is less variable than the capsule-specific binding protein.

StAb3, unlike many other *A. baumannii* phages, is able to infect strains from a broad range of capsule types. We found that StAb3 was unable to infect strains with an insertion or mutation in the genes predicted to synthesize a ManNAc-containing exopolysaccharide in *A. baumannii*, which we have termed PGR. The high penetrance of the pathway combined with its occasional loss, suggests that *Acinetobacter* must balance the benefit of this exopolysaccharide with the strong selective pressure imposed by StAb3-like phages. It is tempting to speculate that StAb3 binds to PGR, and facilitated by its long tail, uses it as a guide through the capsule to reach the cell membrane. Interestingly, although the machinery for PGR synthesis is conserved among *A. baumannii* clinical isolates, the role and structure of this glycan remains unexplored. Further, a similar locus encoding an uncharacterized ManNAc-based polysaccharide serves as the receptor for N4 phage in *E. coli* [65,66]. More work is required to elucidate if these loci, which are also present in other species, are responsible for the synthesis of the same or different ManNAc-containing polysaccharides and to characterize its role in *A. baumannii* lifestyle and virulence. The StAb3 genome has over 75% similarity to that of unclassified phages EAb13, Mystique, and vB_AbaS_TCUP2199, all of which have been characterized as having a broad host range [32–34]. Our analysis of PGR carriage reveals a strong correlation between the host range of these phages with an intact PGR locus, suggesting that they too attach to PGR. Both Mystique and StAb3 display impaired ability, but not complete inability, to infect acapsular strains. Several hypotheses can account for the impaired infection of StAb3 to acapsular strains. It is possible that the capsule stabilizes the phage tail in the proper conformation to enable injection of DNA, or perhaps orients PGR relative to the cell surface. Alternatively, cross talk between capsule and exopolysaccharide receptors may occur. More work is needed to understand if our findings apply to other similar phages and how this exopolysaccharide interacts with bacterial capsule and phage infection.

Many phage therapy cocktails are assembled without knowledge of receptor usage of the phages included in the cocktail, leading to rapid resistance emerging in the bacterial host. There is increasing interest in rational design of phage cocktails using complementary phages with distinct receptors to target bacteria such as *Klebsiella pneumonia* and *Pseudomonas aeruginosa* [28–30]. Further, studies in *A. baumannii* have demonstrated the benefit of combining capsule-dependent phages with capsule-restricted phages [20,67,68]. We are furthering this effort, demonstrating that combining capsule-dependent phages like StAb1 or Bhz16 with CarO-dependent capsule-restricted phages like StAb2 and Bhz15 can prevent or delay the emergence of escape mutants. This highlights the value in identifying phage receptors and applying this information to the rational design of phage cocktails. While several combinations of phages reduce bacterial resistance, not all combinations are equally or consistently effective, which demonstrate that there are still many nuances to phage infection that remain to be discovered. When assessing the efficacy of phage cocktails, we treated bacteria with both phages simultaneously; however, previous work has shown that development of resistant mutants was different if phage cocktails were applied sequentially versus simultaneously [73]. The impact of sequential application of capsule-dependent and capsule-restricted phages is another area for future exploration. We speculate that cellular phage defense pathways also play a role in some cases. It is also likely that some of these phages may carry additional receptor binding proteins. Our detailed characterization of receptor usage will now open the door to rational design of phage therapy cocktails that include phages with different receptors, thereby preventing the rapid emergence of bacterial escape mutants.

## Materials and methods

### Bacterial strains and growth conditions

Bacterial strains used in this study are listed in S7 Table. Unless otherwise noted, strains were grown on LB-agar plates containing LB broth (Fischer BioReagents, BP1427–2) and 1.5% agar (Fisher Bioreagents, BP1423–2) at 37°C. Colonies from these plates were used to inoculate cultures in LB broth grow at 37°C and shaking at 200rpm. When appropriate, LB broth and LB-agar plates were supplemented with 10 µg/mL chloramphenicol, 50 µg/mL apramycin, 100 µg/mL ampicillin, 50 µg/mL kanamycin, 50 µg/mL zeocin, and/or 300 µg/mL hygromycin B.

### Phage isolation

StAb1, StAb2, and StAb3 were isolated from wastewater based upon an adaptation of previously used methods [74]. In brief, wastewater samples were centrifuged at 2,300 x g for 8 minutes at 4°C, then supernatants were filtered through a Millex GS 0.22µm filter (Merck Millipore, SLGSR33SB) to remove any bacteria and particulate matter. Potential bacteriophages were enriched by combining 900µL of 2x LB broth, 100µL of an overnight culture of the isolation host bacterium, and 500µL of the filtered wastewater sample and incubating with shaking overnight at 37°C. Enrichments were centrifuged at 1,700 x g and filtered through a 0.22µm filter to remove any bacteria. 5–10µL of each sample was spotted onto a top agar plate prepared as described below. The bacteriophage titer for any samples which resulted in clearance of bacteria was determined utilizing the plaque assay described below. To ensure the isolate contained only one bacteriophage, a 10µL pipette tip was stabbed into an isolated plaque, swirled in a mixture of 180 µL of LB broth + 20 µL of an overnight culture of the isolation host bacterium and incubated with shaking at 37°C for approximately 6 hours. This sample was centrifuged and filtered to remove any bacteria as described below and plated on top agar plates with the isolation host bacterium. The isolation process was repeated for a total of three times to ensure isolation of a single clonal bacteriophage.

### Phage propagation

All phage strains utilized in this study are listed in S8 Table along with details about their isolation and propagation host bacteria. To propagate the phages, 10 mL of LB broth (with 10 mM MgCl_2_ for propagation of StAb2) was inoculated with 100 µL of an overnight culture of the propagation host bacterium and grown shaking until the OD_600_ reached approximately 0.4 at 37°C. Then, 100 µL of the previous phage stock was added to this culture (at an MOI of approximately 0.1) and incubation continued. Following clearing or overnight incubation, samples were centrifuged at 6,200 x g for 8 minutes and the supernatant was filtered through a 0.22 µm filter to remove any remaining bacteria. Bacteriophage lysates were stored at 4°C for utilization in future experiments.

### Plaque assays

Double layer plaque assays were used to determine bacteriophage titers as well as assess the capacity of bacteriophages to infect specific hosts. A top agar was prepared with 4 mL of molten LB with 0.4% agar combined with 25–200 µL of an overnight culture of the bacterial strain of interest and poured over an LB-agar plate. Phage lysates were serially diluted 10-fold in LB 8 times and 2 µL of each dilution were spotted onto the top agar plates and incubated for 6–20 hours at 30°C for the host range analysis (Fig 1D) and 37°C for all other assays. We found that StAb3 is only able to consistently form visible plaques when using a lower percentage of top agar and incubated at lower temperatures. Therefore, StAb3 was instead plated on LB top agar plates prepared with 0.2% agar and 25µL of an overnight culture of the bacterial strain of interest and poured over an LB-agar plate. These plates were incubated at room temperature (~23°C). Additionally, for both StAb2 and StAb3, using well hydrated plates by minimizing drying time for top-agar plates drastically improved our ability to consistently visualize plaques. Following incubation, plates were observed for plaques and areas of clearance. If individual plaques were observed, they were counted in the most concentrated dilution that was still countable and used to calculate PFU/mL. For some combinations of phage and bacteria, in spots where the phage was highly concentrated, the entire spot cleared showing evidence of lysis; however, as the phage was diluted, it never formed individual plaques. In the host range analysis, when this occurred, the least concentrated spot that still had clearance was noted, and as a proxy for a PFU/mL count was assumed to have the equivalent of 20 individual plaques and used to calculate PFU/mL, and when the data was reported, are marked with a black dot. For all other plaque assays, when this occurred, it was noted, PFU/mL was reported as the limit of detection (5000 PFU/mL), and they are marked with c indicating clearance. For the other strain and bacteria combinations, no clearance was observed at any dilution, PFU/mL was reported as the limit of detection (5000 PFU/mL), and they are marked with nd indicating that phage lysis was not detected. All plaque assays were performed with three independent biological replicates.

### Transmission electron microscopy

For analyses of phages at the ultrastructural level, samples were allowed to absorb onto freshly glow discharged formvar/carbon-coated copper grids (200 mesh, Ted Pella Inc., Redding, CA) for 10 min. Grids were then washed two times in dH2O and negative stained with 1% aqueous uranyl acetate (Ted Pella Inc.) for 1 min. Excess liquid was gently wicked off and grids were allowed to air dry. Samples were viewed on a JEOL 1200EX transmission electron microscope (JEOL USA, Peabody, MA) equipped with an AMT 8 megapixel digital camera (Advanced Microscopy Techniques, Woburn, MA).

### Phage sequencing and genomic analysis

A 1 mL sample of phage lysate was treated with 10 µL (20 units) of Turbo DNase (Thermo Fischer Scientific) and 100 µL of TURBO DNase Buffer, incubating at 37°C for 30 minutes to remove any bacterial DNA. DNase was inactivated by adding EDTA to a final concentration of 15mM and heating at 75°C for 10 minutes. DNA was then purified utilizing a Phage DNA Isolation Kit (Norgen Biotek, SKU 46800). Purified DNA was prepared for Illumina sequencing utilizing Nextera Tagmentation reagents, as described previously [75]. Sequencing was performed at the Washington University Center for Genome Sciences and Systems Biology DNA Sequencing Innovation Lab following standard procedures on an Illumina MiniSeq, 2x150 paired-end, resulting in ~50,000–150,000 reads per genome.

Demultiplexed reads were processed utilizing Trimmomatic 0.39 to remove adapter sequences, then genomes assembled with SPAdes 4.0.0 with default parameters [76,77]. Genomes were assessed using Bandage to confirm purity and circular permutation, indicating completeness of the genome sequence [78]. Genomes were manually reordered and reoriented to maximize synteny with closest relatives, as identified through BLAST searches of the complete genomes [79]. Whole-genome phylogenetic trees based on protein sequences were generated using VIPTree [35]. Tree was rendered using SplitsTree App 6.0.0; the Show Trees method was used (default options) so as to obtain a Tree View visualization [80]. Taxonomic assignment was performed using taxMyPhage web version 3.3.6 [81]. Genomes were annotated using Pharokka 1.7.3 and Phold 0.2.0, with default parameters [82–86]. Clinker 0.0.31 was used to generate phage genome comparison maps [87].

Putative depolymerases of StAb2 were identified using PhageDPO Galaxy version 0.1.0 and DePP web version 1.0.0 utilizing Pharokka-annotated ORFs [55,88]. Homologues of putative depolymerases were identified in other *Lazarusvirus* and *Hadassahvirus* isolates via BLASTP. Multiple sequence alignments were then generated using Clustal Omega on the EMBL-EBI Job Dispatcher [89,90].

### Density gradient

A silica-based density gradient was utilized to semi-quantify capsule production using a modified version of previously published protocols [38,91]. Bacterial strains of interest were streaked onto an LB-agar plate and incubated for 16 hours at 37°C. Bacteria was scraped from the plate with a pipette tip and resuspended in PBS (Gibco, 70011–004) and normalized to an OD_600_ of 3. 1 mL of bacteria in PBS was centrifuged for 5 minutes at 5,000 x g in a 1.5mL microcentrifuge tube, and the supernatant was removed. The pellet was resuspended in 675 µL of PBS, then 325 µL of LUDOX HS-30 colloidal silica (Sigma-Aldrich, 420824) was added and mixed by inversion. The mixture was centrifuged for 30 minutes at 9000 x g. The microcentrifuge tubes were photographed and the distance from the bottom of the microcentrifuge tube to the band of bacteria was measured.

### Acquisition of capsule mutant *A. baumannii*

The plasmids and primers used in this study are listed in S9 and S10 Tables respectively. The capsule mutant for UPAB1 was generated by removing the *wzy* gene by allelic exchange as previously described [92,93]. Briefly, an approximately 300 bp region upstream and downstream of *wzy* was amplified from UPAB1 genomic DNA. These regions were fused to an FRT site-flanked apramycin resistance cassette from pKD4-Apr using overlap extension PCR. This DNA was electroporated into UPAB1 carrying pAT04, a plasmid that encodes a RecAB recombinase and a hygromycin B resistance cassette followed by selection on LB-agar with apramycin. The strain was cured of pAT04 by serially passaging, isolating colonies and patching on LB-agar and LB-agar with hygromycin B until an isolate was identified that was no longer resistant to hygromycin B. The apramycin cassette was then removed by introducing the plasmid pAT03, which encodes an IPTG-inducible FLP recombinase and a hygromycin B resistance cassette, then plating on LB-agar with hygromycin B and 2mM IPTG. Successful removal was verified by confirming a lack of growth on LB-agar with apramycin and PCR amplification of the deleted region. The strain was cured of the pAT03 plasmid by passaging, plating for individual colonies, and patching on LB and hygromycin B to identify an isolate which is no longer resistant to hygromycin B.

The capsule mutants of Ab5075, AbCAN2, and Ab014 were acquired spontaneously. Upon streaking freezer stocks of these strains on an LB-agar plate for isolation of individual colonies, two morphologies were observed, a primary morphology that is more opaque and mucoid and another, less frequent morphology that is more translucent and non-mucoid. It was predicted that the mucoid colonies were the wild-type capsulated strains while the non-mucoid colonies had lost their capsule. These two colony types were isolated and assessed for capsule production by the density gradient described above. All of the capsule mutants were further validated by sequencing as described below. K-locus type for each wild-type strain was identified using Pathogenwatch [24,94].

### Generation of UPAB1 Δ*wzy* transposon mutagenesis library

A transposon mutant library in UPAB1 Δ*wzy* was prepared using a modified version of vector pJNW684 which encodes the Himar1 mariner transposon system as described previously [95,96]. Plasmids and primers used in this study are listed in S9 and S10 Tables respectively. The selection marker of pJNW684 is originally a kanamycin resistance cassette; however, many *A. baumannii* strains, including UPAB1 are resistant to this antibiotic; therefore, it was exchanged for an apramycin resistance cassette. The apramycin resistance cassette was amplified from pUCT18T-miniTn7T-Apr, then introduced into pJNW684 using NEBuilder HiFi DNA Assembly Master Mix (New England Biolabs). The transposon was introduced into UPAB1 Δ*wzy* through conjugation from *E. coli* S17-1 λ-pir carrying pJNW684-Apr. Conjugation was facilitated by the conjugal helper plasmid pRK2013 carried in *E. coli* HB101 and transposon insertion was facilitated by the plasmid pTNS2 carried by *E. coli* 100D. UPAB1 Δ*wzy* and each *E. coli* strain were grown overnight in LB at 37°C in the appropriate antibiotic. 35 mL of each strain was centrifuged at 6500 rpm for 3 minutes and washed once with LB, centrifugating at 6500 rpm for 3 minutes. The pellets of all bacteria were resuspended in 1.4 mL of LB and combined, and 20uL of this bacteria mixture was spotted 70 times on LB-agar plates for conjugation to occur. Plates were dried for 30 minutes at room temperature, then incubated at 37°C for 1 hour. To select for UPAB1 Δ*wzy* isolates with transposons inserted, the bacterial spots were resuspended in LB and spread on LB-agar plates with 10 µg/mL chloramphenicol and 50 µg/mL apramycin and incubated overnight at 37°C.

### Selection of StAb2-resistant UPAB1 Δ*wzy* transposon mutants

In order to identify bacterial genes essential for infection by StAb2, UPAB1 Δ*wzy* transposon mutants from the library generated above that are resistant to StAb2 were isolated. 100 mL LB with 60 µg/mL apramycin was inoculated with 100 µL (4,000 CFU) of the transposon mutagenesis library and grown at 37°C shaking, after 1, 3, and 8 hours, 3.5x10^8^ PFU of StAb2 was added, and after 3, 8, and 24 hours, a sample of the culture was taken and plated on LB-agar plates with 60 µg/mL apramycin and 10 µg/mL chloramphenicol to obtain isolated colonies. Five isolates from each timepoint were tested for susceptibility to StAb2 by plaque assay. All isolates were resistant to StAb2. Genomic DNA was purified from one isolate from the 3-hour timepoint, and 2 isolates each from the 8 and 24 hour timepoints using the PureLink Genomic DNA Mini Kit (Thermo Fisher Scientific). DNA was sequenced by Illumina sequencing by SeqCenter and the location of transposon insertion was determined using Geneious Prime 2024.0.7 (https://geneious.com).

### Bacterial growth curves

To assess the influence of treatment with bacteriophages on bacterial growth, growth curves were performed. Solutions containing overnight culture of the indicated bacterium were normalized to an OD_600_ of 0.01 and bacteriophage lysate was added to the indicated MOIs (with an MOI of 1 corresponding to 2x10^6^ PFU/mL). When multiple bacteriophages were used in a single treatment, they were added at equal MOIs to have a total of the reported MOI. For example, if two phages were added at a total MOI of 1, then an MOI of 0.5 for each phage was added. 150 µL aliquots of these solutions were added to the wells of a polystyrene 96-well plate (Corning, 3788). Plates were incubated at 37°C with shaking and OD_600_ was measured every 30 minutes in a microplate reader (Accuris Smartreader 96-T or BioTek Synergy HTX). Each combination of phages and bacteria was performed with three independent biological replicates, each graphed independently. The growth curves in 7C, 7D, and S9A-B were performed using the Accuris Smartreader 96-T (MR9600-T) each with 6 technical replicates shaking at 4.7 Hz. The growth curves in 7E and S9D Replicate 2 were performed with the same settings but with 3 technical replicates. The growth curves in 2A, 3A, S9C, and S9D Replicate 3 were performed using the BioTek Synergy HTX with orbital shaking at 236 cpm and an amplitude of 4mm with 3 technical replicates.

### Generation of escape mutants

398 mutants resistant to StAb1 (398-StAb1eA-H) or StAb2 (398-StAb2eB-H), 17978 Δ*pglC* mutants resistant to StAb2(17978 ∆pglC-StAb2E1-2), and G7 mutants resistant to Bhz16 were acquired as follows. 150 µL aliquots of LB with an overnight culture of the indicated bacterium normalized to an OD_600_ of 0.01 and the bacteriophage of interest were incubated at 37°C in a 96-well plate (Corning, 3788) shaking for 16–24 hours until visibly turbid. Bacteria from the wells were streaked onto an LB-agar plate to obtain isolated colonies. An individual colony was isolated and durable resistance to the bacteriophage of interest was assessed with a plaque assay as described above. When MC47.2 was grown with StAb3 in this manner, bacterial growth occurred; however, when isolates were assessed, they remained susceptible to StAb3. To acquire MC47.2 mutants resistant to StAb3, 4 mL of top agar (LB with 0.4% agar) was combined with 25 µL of overnight culture of the indicated bacterium and poured over LB-agar plates and LB-agar plates supplemented with chloramphenicol. A 10µL drop of StAb3 phage lysate (~5 x 10^9^ PFU/mL) was spotted onto the top agar plates and incubated at 30°C or 20°C until individual colonies appeared in the area of clearance. Colonies were selected and streaked onto a fresh LB-agar plate to isolate individual colonies, and resistance to StAb3 was assessed by plaque assay. The Up280 mutant resistant to StAb3 was acquired in a similar manner; however, 0.2% agar was used for the top-agar instead of 0.4% and plates were incubated at room temperature (~23°C). To acquire one of the 398 mutants resistant to StAb2 (398-StAb2eA), 4 mL of top agar was combined with 25 µL of overnight culture of 398 and poured over LB-agar plates. StAb2 was spotted on this plate, and then plates were incubated at 37°C until colonies appeared in the area of clearance. Colonies were selected and streaked onto a fresh LB-agar plate to isolate individual colonies and resistance to StAb2 was assessed by plaque assay.

### Sequencing and analysis of capsule mutants and phage resistant strains

Genomic DNA was purified from each capsule mutant, escape mutant, and relevant wild-type strains utilizing the PureLink Genomic DNA Mini Kit (Thermo Fisher Scientific). DNA was sequenced by Illumina sequencing by SeqCenter or SeqCoast. The Illumina sequencing reads for sequenced strains were mapped to the assembled genome listed with Geneious Prime 2024.0.7 (https://geneious.com) utilizing the Map to Reference function with the Geneious Mapper at Medium-Low Sensitivity/Fast. Following assembly, the Find High/Low Coverage function was used to find regions with coverage below 4 standard deviations from the mean and the Find variations/SNPs function was used to identify variants with minimum variant frequency set to 0.2, maximum variant P value set to 10^-6^, and minimum strand-bias P-value set to 10^-5^ when exceeding 65% bias finding variants “Inside & Outside CDS” with the “Approximate P-value” calculation method. SNPs identified with variant frequency >70% were reported as a SNP. Areas of the genome with SNPs of variant frequency <70% or coverage below 4 standard deviations from the mean were manually assessed for evidence of rearrangements, insertions, or deletions. When an assembled genome for the wild-type strain or a closely related strain was available, Illumina sequencing from mutants and their associated wild-type strain was aligned to this genome, and the results from the wild-type strain were used to eliminate any spurious calls, accession numbers are listed in S7 Table. For G7, DNA was sent to Plasmidsaurus for Nanopore sequencing and genome assembly, to which all escape mutants were aligned, and Illumina sequencing for the wild-type strain was used to eliminate any spurious calls. For Ab014, sequencing was performed by McGill University and the Genome Quebec Innovation Center with PacBio, to which Illumina sequences of Ab014 and Ab014-cm were aligned and compared. K-locus annotations to characterize the SNPs found in 398 and G7 escape mutants were performed using Kaptive [24].

### Strain construction

Plasmids and primers used in this study are listed in S9 and S10 Tables respectively. The deletion of *carO* from 398 and 398-StAb1eA and *pgrD* from MC47.2 was generated using a pEX-based exchange vector with counterselection as described previously [97,98]. In brief, regions approximately ~1kb upstream and ~1kb downstream of the genes were amplified from the *A. baumannii* genome, with the *carO*-flanking regions amplified from the 398 genome and the *pgrD*-flanking regions amplified from the MC47.2 genome. These regions were cloned into pEX18-Apr with NEBuilder HiFi DNA Assembly Master Mix (New England Biolabs). The plasmids were introduced into their respective *A. baumannii* strain using a tri-parental mating with *E. coli* Stellar Competent Cells carrying the pEX18 vector serving as the donor, and *E. coli* HB101/pRK2013 facilitating conjugation. *A. baumannii* with successful integration of the plasmid were selected for on LB-agar supplemented with apramycin and chloramphenicol. Integration of the plasmid into the expected location was validated by PCR, then strains were plated on LB-agar with no NaCl and 10% sucrose to select for excision of the plasmid. Isolates that had the desired loss of the genes were identified utilizing PCR with primers flanking the gene.

Complementation of *carO* in UPAB1 Δ*wzy* *carO::tn* was conducted by inserting the *carO* gene from two different *A. baumannii* strains into a neutral site in the chromosome with a miniTn7 system as previously described [99,100]. The constructs were generated by amplifying the putative promoter region (~500 bp upstream) along with the open reading frame of the *carO* genes from 19606 and ACICU, which was then inserted into pUCT18T-mini-Tn7T-Zeo with NEBuilder HiFi DNA Assembly Master Mix (New England Biolabs) following cleavage of the plasmid with KpnI and BamHI. The plasmids were introduced into UPAB1 Δ*wzy* *carO::tn* using a four-parental conjugation technique and complemented strains were selected for on LB-agar supplemented with zeocin and chloramphenicol, and insertion was validated by PCR [99,100].

Complementation of *bcsB* and *wecB* was conducted using the same miniTn7 system [99,100]. The constructs were generated by amplifying the open reading frame of the genes from MC47.2, which was then inserted into pUCT18T-mini-Tn7T-Apr with NEBuilder HiFi DNA Assembly Master Mix (New England Biolabs) following linearization of the plasmid by PCR. The plasmids were introduced into their respective strain using a four-parental conjugation technique and complemented strains were selected for on LB-agar supplemented with apramycin and chloramphenicol, and insertion was validated by PCR [99,100].

### Imipenem MIC

Imipenem susceptibility was determined using the 2-fold broth dilution microtiter assay as previously described [101]. A polystyrene 96-well plate (Corning, 3788) was prepared with 2-fold dilutions of imipenem and each well was inoculated with overnight cultures of tested strains normalized to a final OD_600_ of 0.01. Plates were incubated at 37°C with shaking for 16 hours, then final OD_600_ was measured. MICs were defined as the imipenem concentration at which the OD_600_ was < 10% of that of the non-treated control.

### CarO Alignments

Amino acid sequences of CarO proteins from *A. baumannii* strains used in this study were aligned and percent identity determined using Clustal Omega through Geneious Prime 2025.0.2.

### Adsorption assays

Cultures of strains to be assessed for phage adsorption were inoculated from streaks on LB agar plates into LB and cultured overnight at 37°C. The following morning, the indicator strain on which plaquing is performed (Ab014-cm for StAb2 and MC47.2 for StAb3) was inoculated into LB and cultured until mid-late log phase was reached (OD ~ 1; 2–3 hours). 25 μL of this culture was used to seed 4 mL of LB top agar (0.4% agar for StAb2 and 0.2% agar for StAb3) per agar plate and then allowed to solidify. Simultaneously, overnight cultures of assayed strains were diluted 1:100 into 5 mL of LB broth in 125-mL flasks and incubated with shaking at 37°C until early-mid log was reached (OD ~ 0.3-0.6; corresponding to ~5x10^8^ CFU/mL of bacteria). Flasks containing no bacteria, only LB, were treated similarly as a negative control to identify any background carryover of phage. 10^6^ PFU/mL of phage was then added to the flask and allowed to incubate with shaking at 37°C for 10 minutes. Flasks were immediately transferred onto ice to rapidly cool, and samples were kept on ice for the remainder of processing. 500 μL aliquots were transferred to 1.5 mL microcentrifuge tubes and centrifuged to pellet bacteria (17,000 x g, 2 minutes, 4°C). 100 μL aliquots were removed (supernatant), and the pellet was washed in 500 μL fresh, cold LB and pelleted again. The supernatant was removed and the pellet resuspended in 500 μL fresh, cold LB. 100 μL aliquots were removed (pellet). Supernatant and pellet were both 10-fold serially diluted 5 times, and 20 μL spots of each dilution were plated onto the top agar overlay of the indicator strain. For StAb2, these plates were incubated overnight at 30°C within a BD GasPak EZ containing a damp paper towel to maintain sufficient moisture for consistent StAb2 plaquing. For StAb3, these plates were incubated overnight at room temperature (~23°C). Plaques were counted and used to determine percent binding of phage: (PFU/mL of pellet/ (PFU/mL of pellet + PFU/mL of supernatant)) * 100.

### PGR locus analysis

Genes in the predicted PGR locus in *A. baumannii* were compared to genes from other exopolysaccharide synthesis and transport systems. Percent identity between proteins listed in S6 Fig was determined using UniProt align, which makes use of Clustal Omega on the EMBL-EBI Job Dispatcher [89,90,102–104]. Structural alignment of proteins with existing structure predictions through AlphaFold shown in S5 Table was performed using RCSB PDB pairwise structure alignment with the TM-align method [105].

For identification of homologous PGR loci in *A*. *baumannii*, a non-redundant list of *Acinetobacter baumannii* genomes complete to at least the chromosome level was manually constructed, and genomes were downloaded from NCBI and used to build a custom BLAST database [106]. BLASTn was run using default parameters using the complete PGR locus coding sequence from *A. baumannii* 19606 as the query [79,107,108]. BLASTn results were converted into a BED file containing coordinates of each PGR locus in each genome and seqtk was used to extract the corresponding nucleotide sequence of each locus [109]. These PGR loci were then dereplicated and clustered at 90% identity using vsearch to identify and quantify distinct locus compositions across *A. baumannii* isolates [110]. Genbank files of representative PGR loci from each cluster were then downloaded from NCBI and Clinker was used to compare gene organization and composition [87].

For identification of homologous PGR loci in other *Acinetobacter* spp., a similar protocol was followed in which the complete list of *Acinetobacter* genomes from species other than *A. baumannii* in the NCBI Genome database that were complete to at least the chromosome level were downloaded and used for a BLASTn-based search, as above. Presence or absence of the PGR locus in each genome was then manually assessed and used to quantify carriage rates of the PGR locus. The phylogenetic tree of *Acinetobacter* species was generated using GToTree, using the NCBI representative genomes of each *Acinetobacter* sp. included in the BLAST search [111–117]. The tree was visualized using the Interactive Tree of Life [118].

PGR loci were further identified in the MRSN collection of *A. baumannii* clinical isolates and *A. baumannii* isolates tested for StAb3 susceptibility (BioProjects PRJNA545079 and PRJNA1126181, respectively) as described above and all loci were manually inspected to confirm BLASTn-based results and identify the presence of any pseudogenes [119]. The phylogenetic tree of MRSN and other *A. baumanii* clinical isolates tested for StAb3 susceptibility was generated using GToTree and visualized using the Interactive Tree of Life.

### StAb3 planktonic replication

We adapted the method developed by Alseth et al [33] to measure planktonic replication of StAb3. 3 mL of LB was inoculated with 30 µL of an overnight culture of each strain. 10^4^ PFU/mL of StAb3 was then added to each culture, and incubated overnight at 37°C shaking at 200rpm. 30 µL of these cultures were transferred into a fresh tube of three mL of LB (1:100 dilution) for a total of three days. A 1 mL sample of the final cultures were treated with 50 µL of chloroform and centrifuged to pellet bacteria (13,800 x g for 3 minutes). Phage in the supernatant was quantified by serially diluting the sample and plating 20 µL of each dilution onto top-agar plates with 4 mL of 0.2% agar LB and 25 µL of MC47.2.

## Supporting information

S1 Fig Genomic characteristics of novel *Acinetobacter* phages StAb1, StAb2, and StAb3. (A-C) Circularly permuted diagrams of the genomes of StAb1, StAb2, and StAb3, generated with Phold. ORFs are annotated and colored according to PHROG predictions as indicated. Interior tracks indicate the GC content and skew across the genomes. D) Comparison of annotated ORFs in StAb3 with its closest sequenced relatives: TCUP2199, Mystique, and EAb13, generated using Clinker. Phold-annotated ORFs are identified and colored as in (A). Protein percent identity is indicated with the black lines between each gene and its homolog in the adjacent tracks.
(TIF)

S2 Fig StAb2 can infect acapsular strains. (A) Density gradient assay for 17978, 17978∆*pglC*, the complemented strain 17978∆*pglC *+ *pglC* (PRLM2), and the empty vector control 17978∆*pglC* + EV(PWH1266). The area of bacterial biomass is indicated by the red triangle. The height of this band in mm from the bottom of the tube to the center of the bacterial biomass was used as an indicator of bacterial density and therefore capsular content as marked with a red line. (B) Quantification of band height for strains of 17978 as in (A). (C) Quantification of plaque assay with StAb2 on strains from (A) as measured by PFU/mL in a plaque assay. LOD = 3.7. nd = not detected. (D) Wild-type strains and corresponding capsule mutants assessed for capsule via silica-based density gradient experiments. Statistical significance was determined by a t-test, * P < 0.01, ns P > 0.01. (E) Bhz16 phage escape mutant capsule formation assessed via silica-based density gradient experiments and compared with the wild-type (wt) parental strain G7 (gray). B-E) Three independent replicates and their average is presented with error bars representing sd. B, E) Statistical significance was determined by an ANOVA, and each strain was compared to the wt with Dunnet’s multiple comparisons test, * P < 0.05, ns P > 0.05.
(TIF)

S3 Fig Homology between different *A. baumannii* CarO proteins. (A) Alignment of amino acid sequences of CarO from acapsular strains susceptible to StAb2 and the two CarO proteins used for complementation (CarO^19606^ and CarO^ACICU^)**.** (B) The pairwise percent identity comparisons of the CarO proteins in A.
(TIF)

S4 Fig Both CarO and capsule impact StAb2 infection of planktonic 398. A-D) Growth of 398 or the indicated mutant with and without StAb2 at an MOI of 1. Each graph represents the average of three independent replicates each with three technical replicates presented as a bold line, with each technical replicate shown as a narrow line. E) Area under the curve (AUC) calculated for the growth curves in A-D. AUC for each technical replicate is represented by a point. The average is presented, error bars representing standard deviation (sd). Statistical significance was determined by a Brown-Forsythe and Welch’s ANOVA, with treatments compared using Dunnett’s T3 multiple comparisons test, * P < 0.05, ns P > 0.05.
(TIF)

S5 Fig Twarogviruses carry a highly variable predicted depolymerase that may explain host range restriction. (A) The pairwise percent identity comparisons of the top four most strongly predicted depolymerase genes in StAb2 compared to each homolog in the 29 *Hadassahvirus* and *Lazarusvirus* genomes available on NCBI, as well as Bhz15. The protein sequence homolog was compared to every other homolog in the 31 strains using Clustal Omega, and the percent identity of each comparison is shown. (B) A whole-proteome phylogenetic tree of all 29 *Hadassahvirus* and *Lazarusvirus* genomes available on NCBI, as well as StAb2 and Bhz15, generated using VIPTree. Scale indicates genomic distance, as defined previously [35]. (C) Protein sequence-based phylogenetic trees of strongly predicted depolymerase CDS_0239 from StAb2 and its homologs, generated using Clustal Omega. (D) A diagram of CDS_0239 from StAb2, with functional domains annotated. A comparison of the protein sequence among homologs from Twarogviruses is displayed, revealing two short linker domains (aa 1–68 and aa 977–1024), with two larger highly variable domains (aa 69–976 and 1025–1257). The pairwise percent identities from Clustal Omega protein alignments are shown via heatmaps for each domain. Taxonomy of each source genome is indicated along the edge of the heatmap.
(TIF)

S6 Fig Homology between proteins involved in EPS biosynthesis and secretion pathways. The pairwise percent identity comparisons of the proteins identified as having homology in Fig 5C. PgrA (A), PgrC (B), PgrD (C), PgrE (D), PgrF (E), PgrG (F).
(TIF)

S7 Fig PGR loci comparison. Representatives of each PGR locus cluster as identified by vsearch (at 90% identity) and aligned by Clinker. The *pgr* genes are colored as indicated, as are any homologous genes present in more than one of the representative sequences. Labels at left indicate the number of unique genomes whose PGR locus clustered with the representative sequence and the name of the *A. baumannii* genome that is the source of the representative sequence used for alignment.
(TIF)

S8 Fig StAb3 can infect other *Acinetobacter* strains. Quantification of plaque assay with StAb3 on the listed non-*baumannii Acinetobacter* strains. LOD = 3.7. nd = not detected. c = areas of clearing, but no individual plaques, observed.
(TIF)

S9 Fig Combinations of phages prevent or delay bacterial resistance. Growth of 398 (A and C) or G7 (B and D) with the indicated individual phage or phages combined. Phages were added to an MOI of 1. Each graph represents a single independent replicate with the average of three (A-B) or six (C-D) technical replicates presented as a bold line, with each technical replicate shown as a narrow line.
(TIF)

S10 Fig Combinations of phages prevent or delay bacterial resistance AUC quantification. Area under the curve (AUC) calculated for growth curves of 398 (A, C) or G7 (B, D) with the indicated individual phage or phages combined from Figs 7 and S9. Each graph represents three independent replicates each with three (C-D) or six (A-B) technical replicates, with each point representing a single technical replicate. (A) corresponds to Figs 7C and S9A, (B) corresponds to Figs 7D and S9B, (C) corresponds to S9C Fig, and (D) corresponds to Figs 7E and S9D. The average is presented, error bars representing standard deviation (sd). Statistical significance was determined by a Brown-Forsythe and Welch’s ANOVA, with treatments compared using Dunnett’s T3 multiple comparisons test, * P < 0.05, ns P > 0.05.
(TIF)

S1 Table Capsule mutant confirmation sequencing analysis. Location and type of SNPs identified in spontaneous capsule mutants. Predicted insertions or rearrangements are reported as I/R.
(PDF)

S2 Table Phage-resistant mutant sequencing analysis. Location and type of SNPs identified in phage-resistant mutants. Predicted insertions or rearrangements are reported as I/R.
(PDF)

S3 Table Capsule types of wild-type *A. baumannii* strains. K-locus and capsular polysaccharide types and structure of *A. baumannii* strains determined using Kaptive [24].
(PDF)

S4 Table Imipenem MIC. Susceptibility of CarO mutants to imipenem based upon minimum inhibitory concentration (MIC).
(PDF)

S5 Table Structural alignment of exopolysaccharide synthesis and transport proteins. Structural alignment of a subset of exopolysaccharide synthesis and transport proteins described in Fig 5C-D using RCSB PDB pairwise structure alignment [105].
(PDF)

S6 Table Description of PGR locus present in strains tested for StAb3-like phage susceptibility. All strains with published Mystique or EAb13 host range, or strains for which we measured StAb3 host range, were analyzed for the presence and status of the PGR locus. Summary data is provided at the bottom of the table. See also Fig 6A for a graphical summary [32,33].
(PDF)

S7 Table Bacterial Strains. List of bacterial strains used in the paper.
(PDF)

S8 Table Bacteriophages. List of all bacteriophages with their isolation and propagation hosts used in this paper.
(PDF)

S9 Table Plasmids. List of all plasmids used in the paper.
(PDF)

S10 Table Primers. List of all primers used in the paper.
(PDF)
