# Supplementary figures and images for: Acinetobacter phages use distinct strategies to breach the capsule barrier
Source: PLoS Pathog. 2025 Sep 29;21(9):e1013536. doi: 10.1371/journal.ppat.1013536 (PMC12507263; doi:10.1371/journal.ppat.1013536)

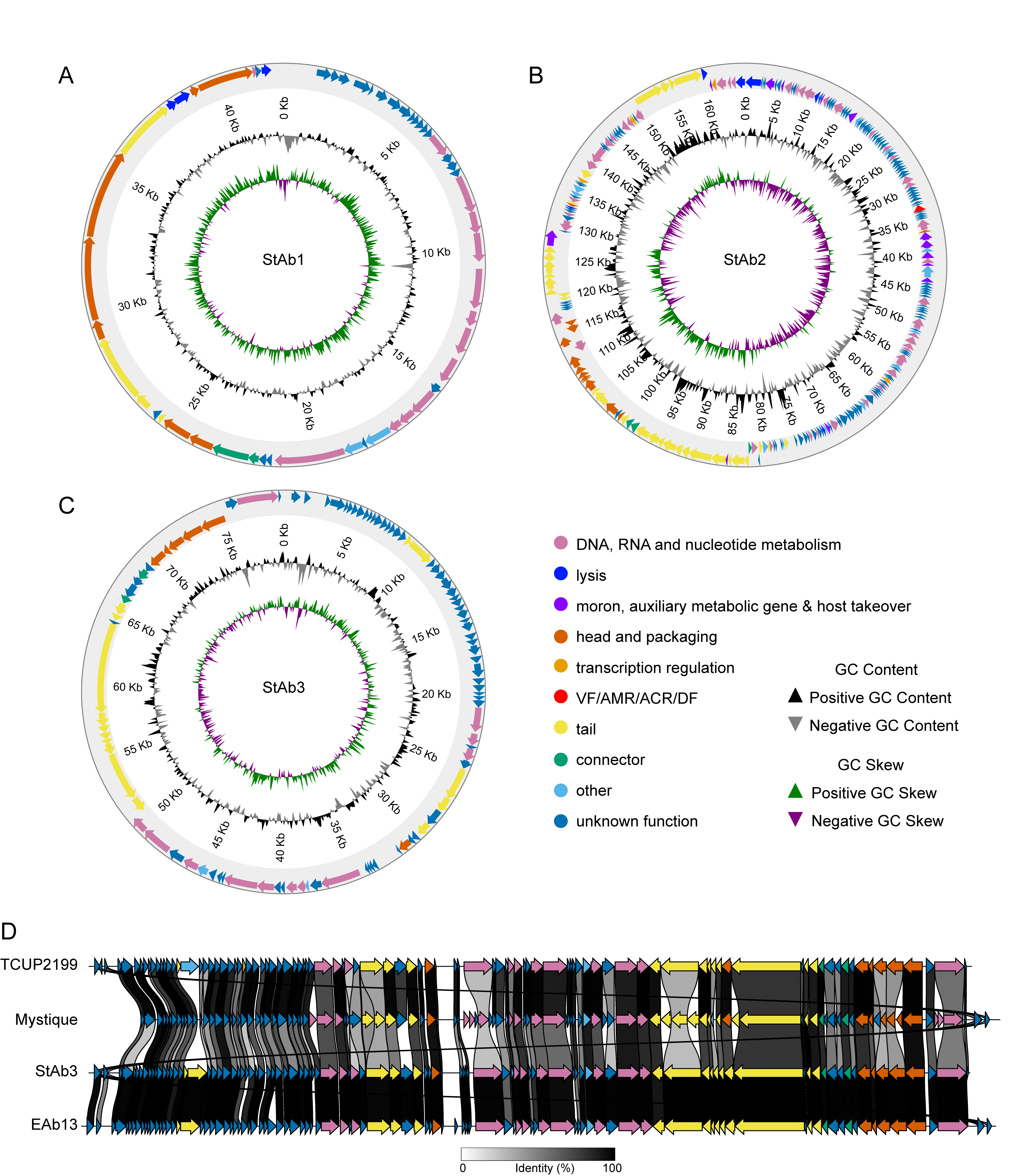

Supplement: S1 Fig — (A-C) Circularly permuted diagrams of the genomes of StAb1, StAb2, and StAb3, generated with Phold. ORFs are annotated and colored according to PHROG predictions as indicated. Interior tracks indicate the GC content and skew across the genomes. D) Comparison of annotated ORFs in StAb3 with its closest sequenced relatives: TCUP2199, Mystique, and EAb13, generated using Clinker. Phold-annotated ORFs are identified and colored as in (A). Protein percent identity is indicated with the black lines between each gene and its homolog in the adjacent tracks. (TIF) [file ppat.1013536.s001.tif]

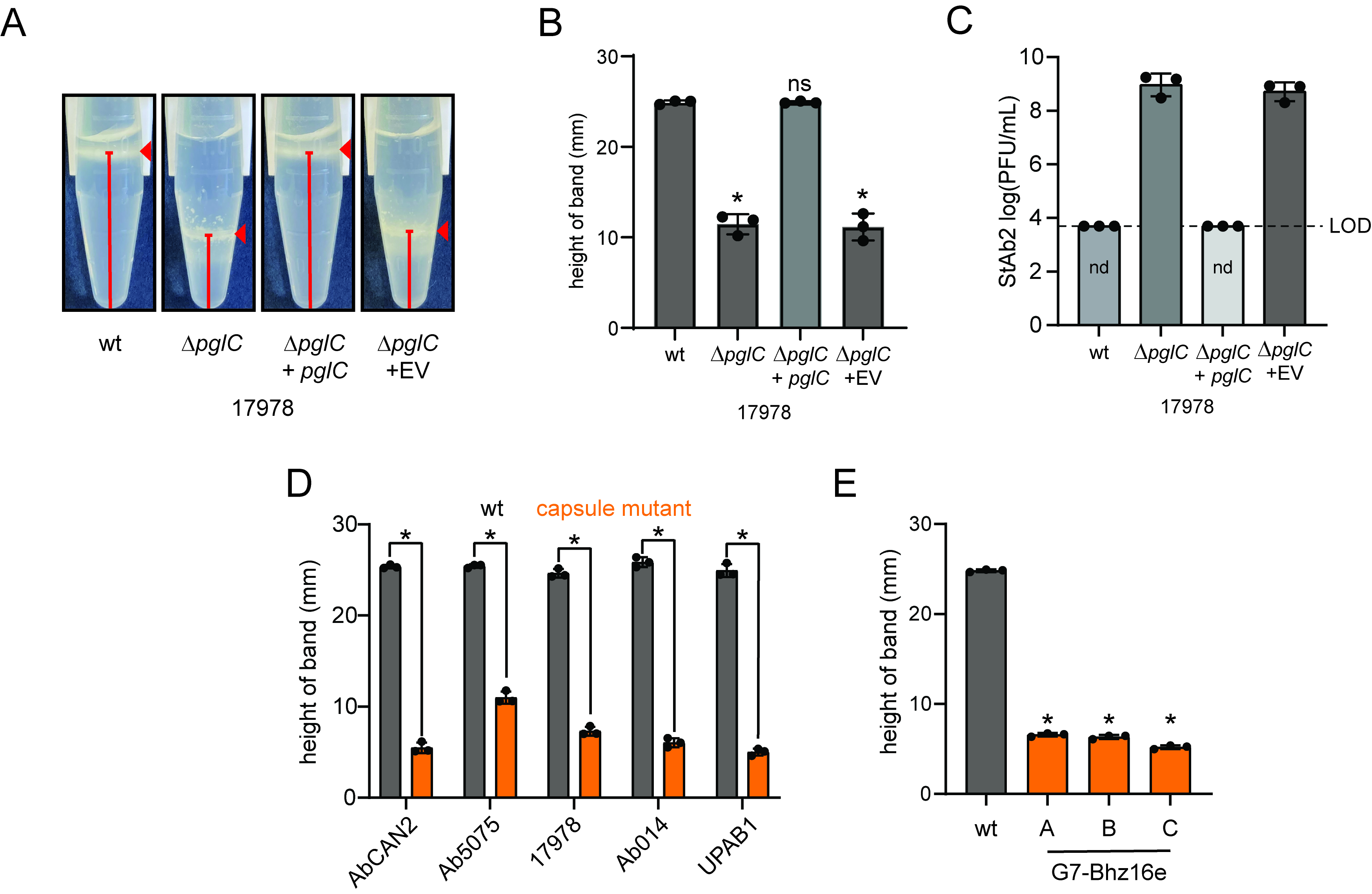

Supplement: S2 Fig — (A) Density gradient assay for 17978, 17978∆pglC, the complemented strain 17978∆pglC + pglC (PRLM2), and the empty vector control 17978∆pglC + EV(PWH1266). The area of bacterial biomass is indicated by the red triangle. The height of this band in mm from the bottom of the tube to the center of the bacterial biomass was used as an indicator of bacterial density and therefore capsular content as marked with a red line. (B) Quantification of band height for strains of 17978 as in (A). (C) Quantification of plaque assay with StAb2 on strains from (A) as measured by PFU/mL in a plaque assay. LOD = 3.7. nd = not detected. (D) Wild-type strains and corresponding capsule mutants assessed for capsule via silica-based density gradient experiments. Statistical significance was determined by a t-test, * P < 0.01, ns P > 0.01. (E) Bhz16 phage escape mutant capsule formation assessed via silica-based density gradient experiments and compared with the wild-type (wt) parental strain G7 (gray). B-E) Three independent replicates and their average is presented with error bars representing sd. B, E) Statistical significance was determined by an ANOVA, and each strain was compared to the wt with Dunnet’s multiple comparisons test, * P < 0.05, ns P > 0.05. (TIF) [file ppat.1013536.s002.tif]

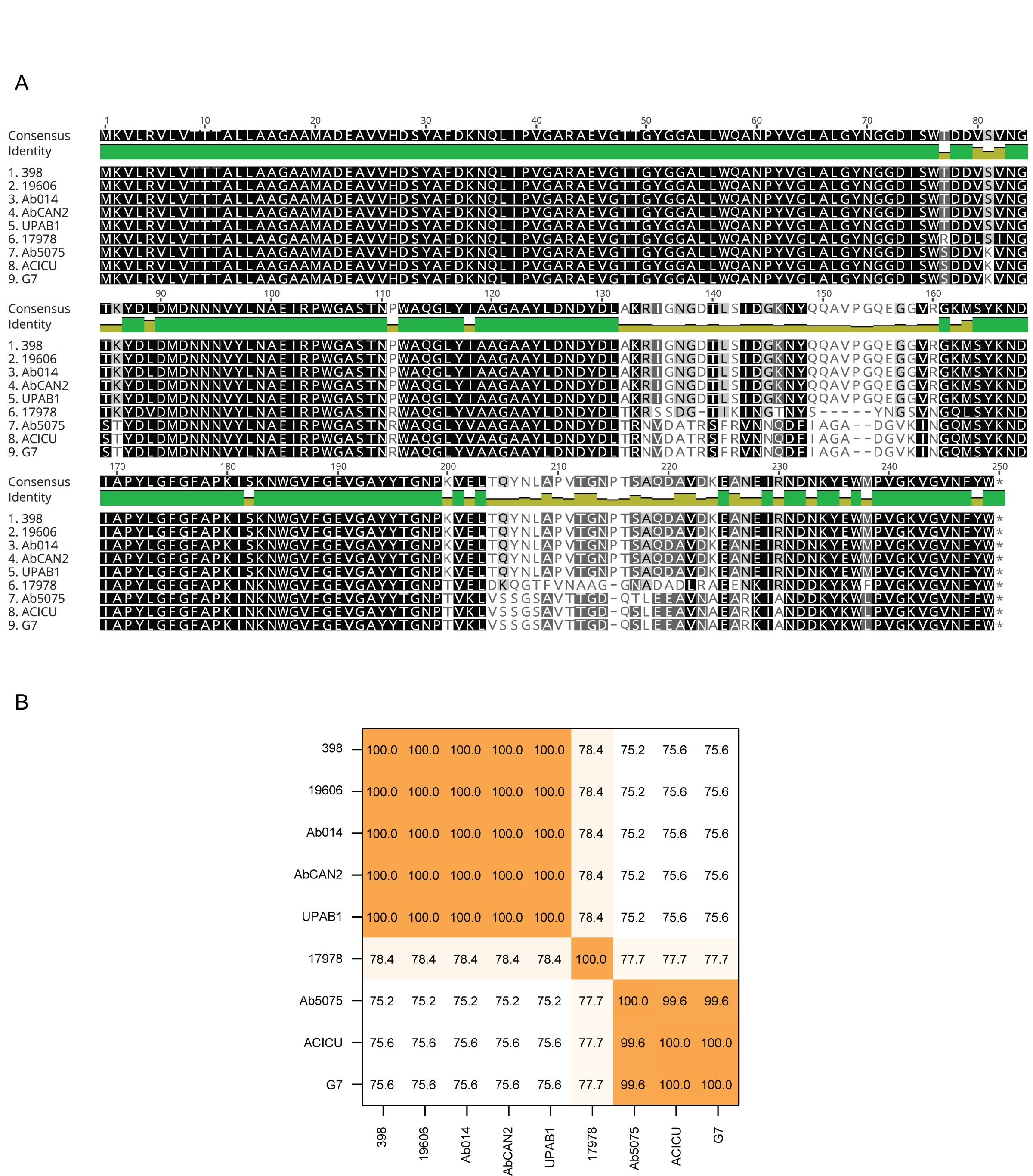

Supplement: S3 Fig — (A) Alignment of amino acid sequences of CarO from acapsular strains susceptible to StAb2 and the two CarO proteins used for complementation (CarO19606 and CarOACICU). (B) The pairwise percent identity comparisons of the CarO proteins in A. (TIF) [file ppat.1013536.s003.tif]

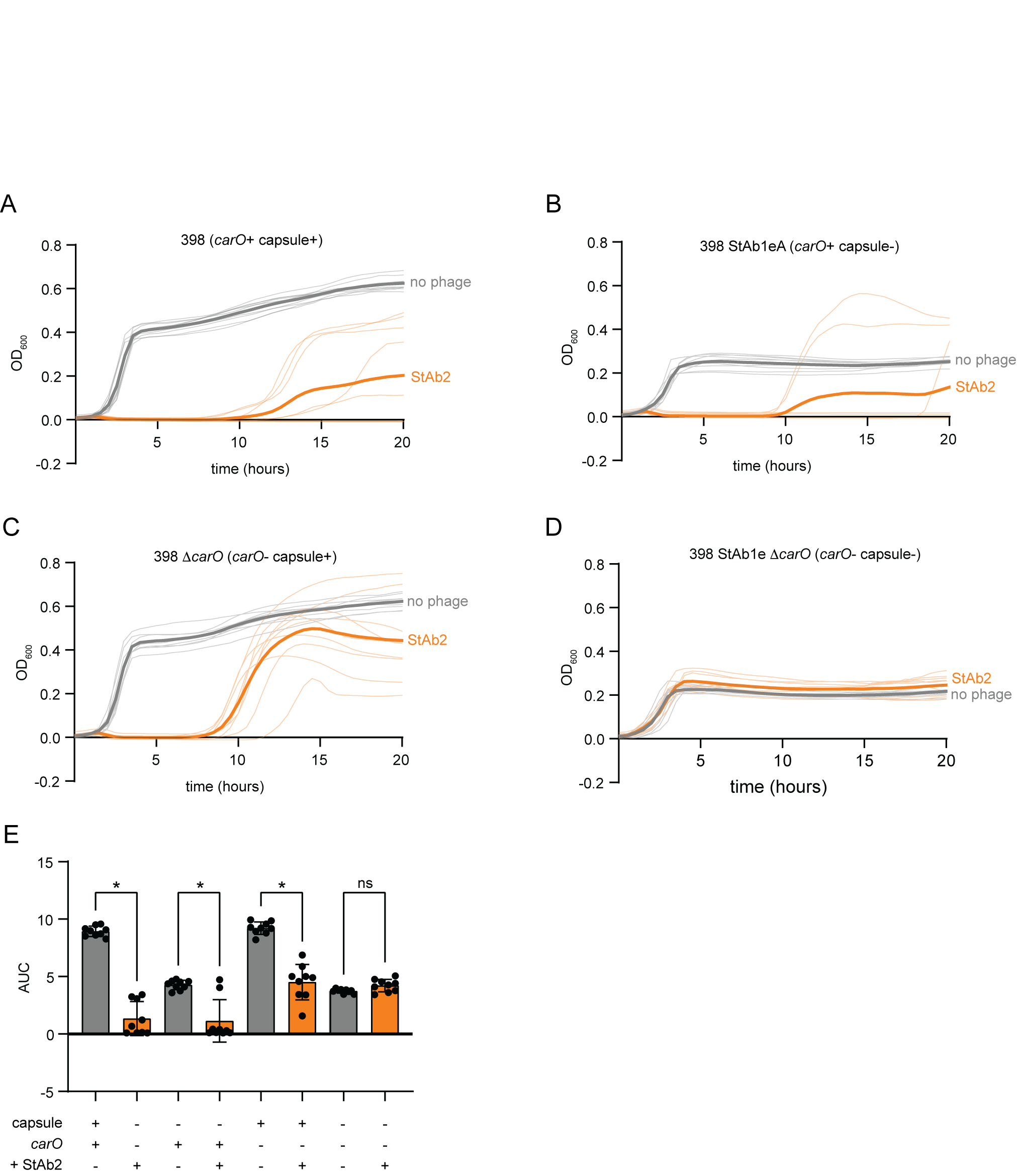

Supplement: S4 Fig — A-D) Growth of 398 or the indicated mutant with and without StAb2 at an MOI of 1. Each graph represents the average of three independent replicates each with three technical replicates presented as a bold line, with each technical replicate shown as a narrow line. E) Area under the curve (AUC) calculated for the growth curves in A-D. AUC for each technical replicate is represented by a point. The average is presented, error bars representing standard deviation (sd). Statistical significance was determined by a Brown-Forsythe and Welch’s ANOVA, with treatments compared using Dunnett’s T3 multiple comparisons test, * P < 0.05, ns P > 0.05. (TIF) [file ppat.1013536.s004.tif]

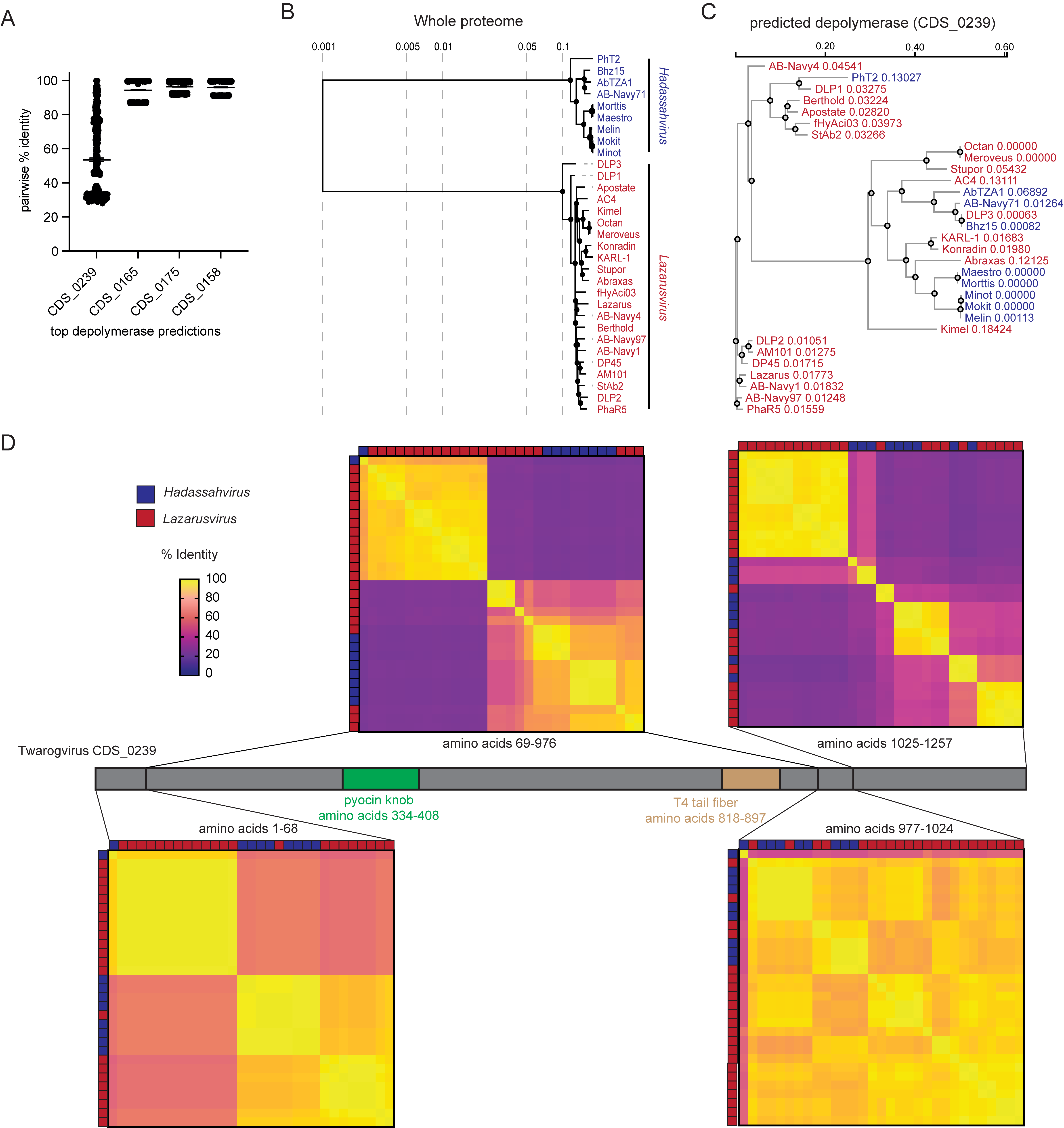

Supplement: S5 Fig — (A) The pairwise percent identity comparisons of the top four most strongly predicted depolymerase genes in StAb2 compared to each homolog in the 29 Hadassahvirus and Lazarusvirus genomes available on NCBI, as well as Bhz15. The protein sequence homolog was compared to every other homolog in the 31 strains using Clustal Omega, and the percent identity of each comparison is shown. (B) A whole-proteome phylogenetic tree of all 29 Hadassahvirus and Lazarusvirus genomes available on NCBI, as well as StAb2 and Bhz15, generated using VIPTree. Scale indicates genomic distance, as defined previously [35]. (C) Protein sequence-based phylogenetic trees of strongly predicted depolymerase CDS_0239 from StAb2 and its homologs, generated using Clustal Omega. (D) A diagram of CDS_0239 from StAb2, with functional domains annotated. A comparison of the protein sequence among homologs from Twarogviruses is displayed, revealing two short linker domains (aa 1–68 and aa 977–1024), with two larger highly variable domains (aa 69–976 and 1025–1257). The pairwise percent identities from Clustal Omega protein alignments are shown via heatmaps for each domain. Taxonomy of each source genome is indicated along the edge of the heatmap. (TIF) [file ppat.1013536.s005.tif]

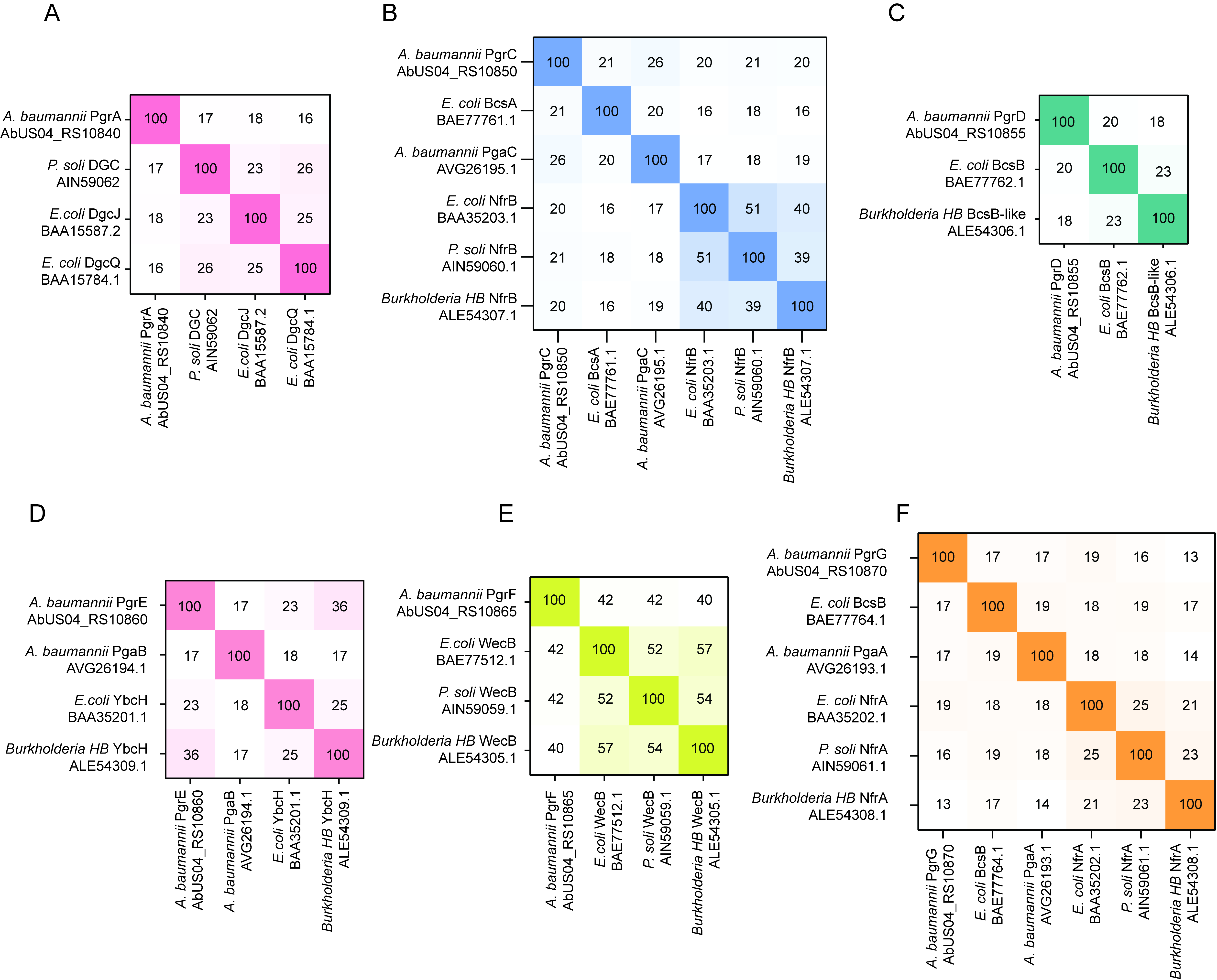

Supplement: S6 Fig — The pairwise percent identity comparisons of the proteins identified as having homology in Fig 5C. PgrA (A), PgrC (B), PgrD (C), PgrE (D), PgrF (E), PgrG (F). (TIF) [file ppat.1013536.s006.tif]

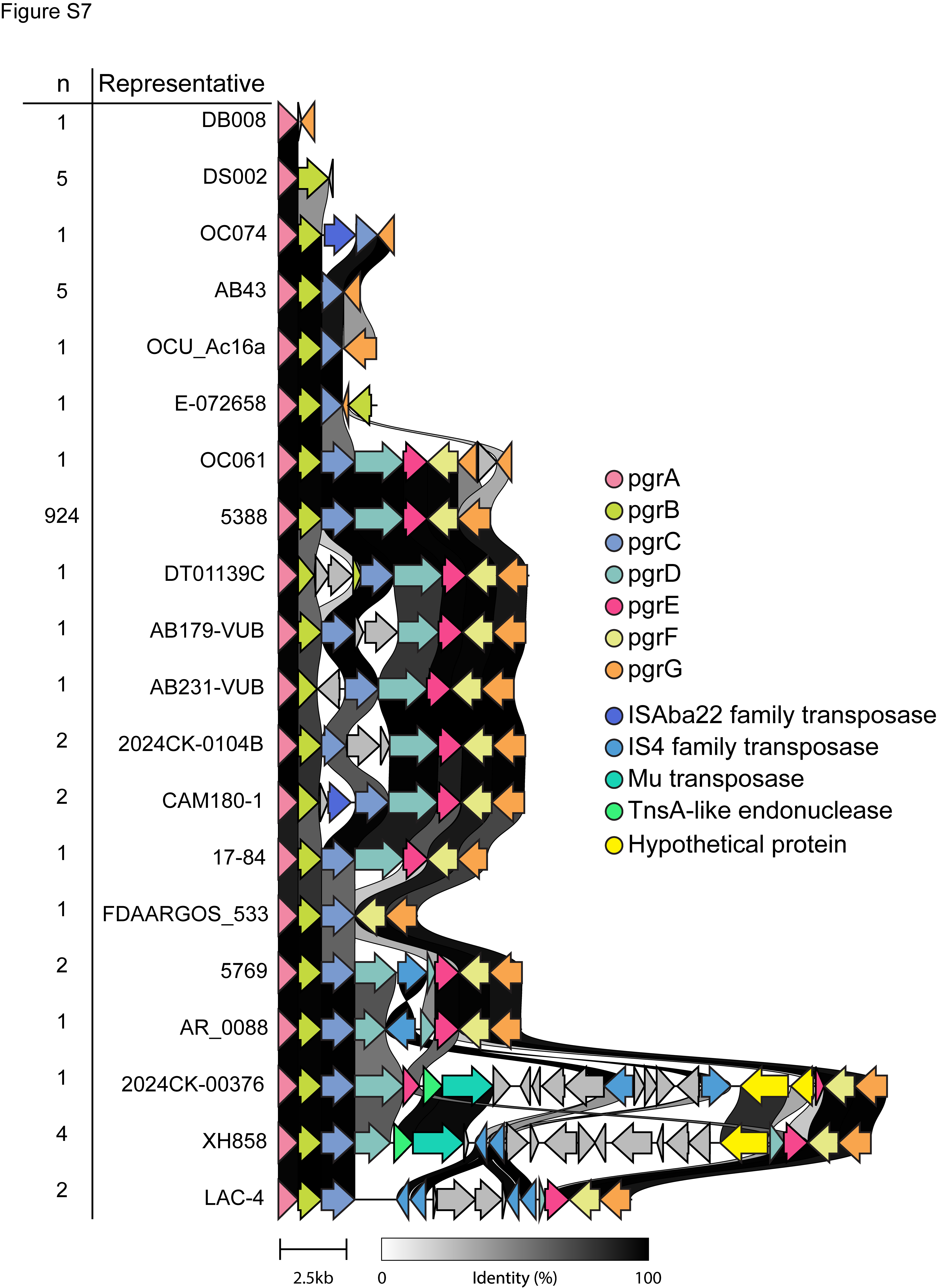

Supplement: S7 Fig — Representatives of each PGR locus cluster as identified by vsearch (at 90% identity) and aligned by Clinker. The pgr genes are colored as indicated, as are any homologous genes present in more than one of the representative sequences. Labels at left indicate the number of unique genomes whose PGR locus clustered with the representative sequence and the name of the A. baumannii genome that is the source of the representative sequence used for alignment. (TIF) [file ppat.1013536.s007.tif]

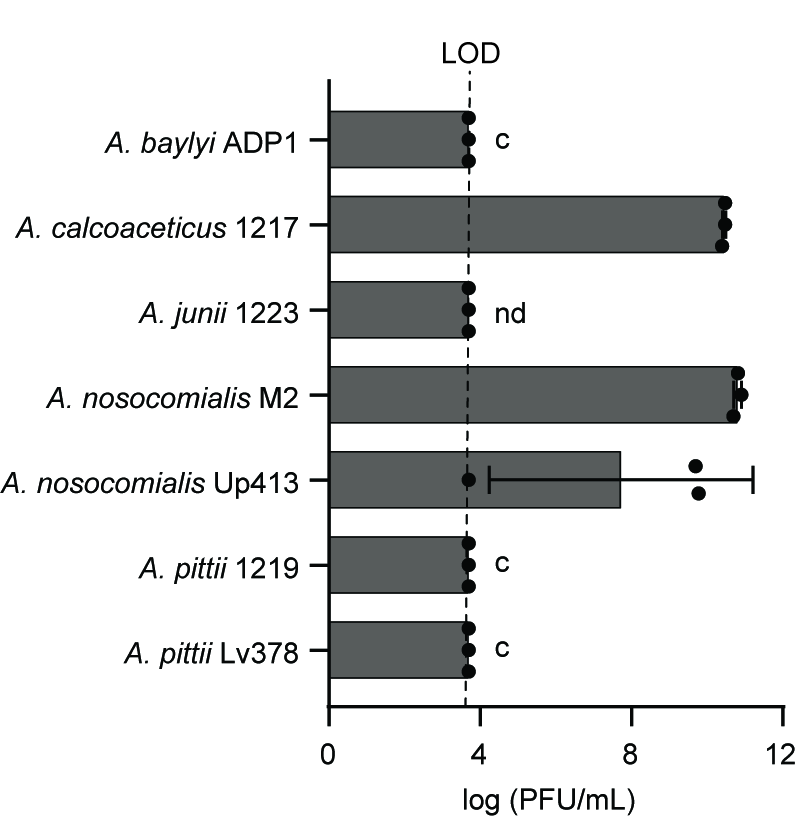

Supplement: S8 Fig — Quantification of plaque assay with StAb3 on the listed non-baumannii Acinetobacter strains. LOD = 3.7. nd = not detected. c = areas of clearing, but no individual plaques, observed. (TIF) [file ppat.1013536.s008.tif]

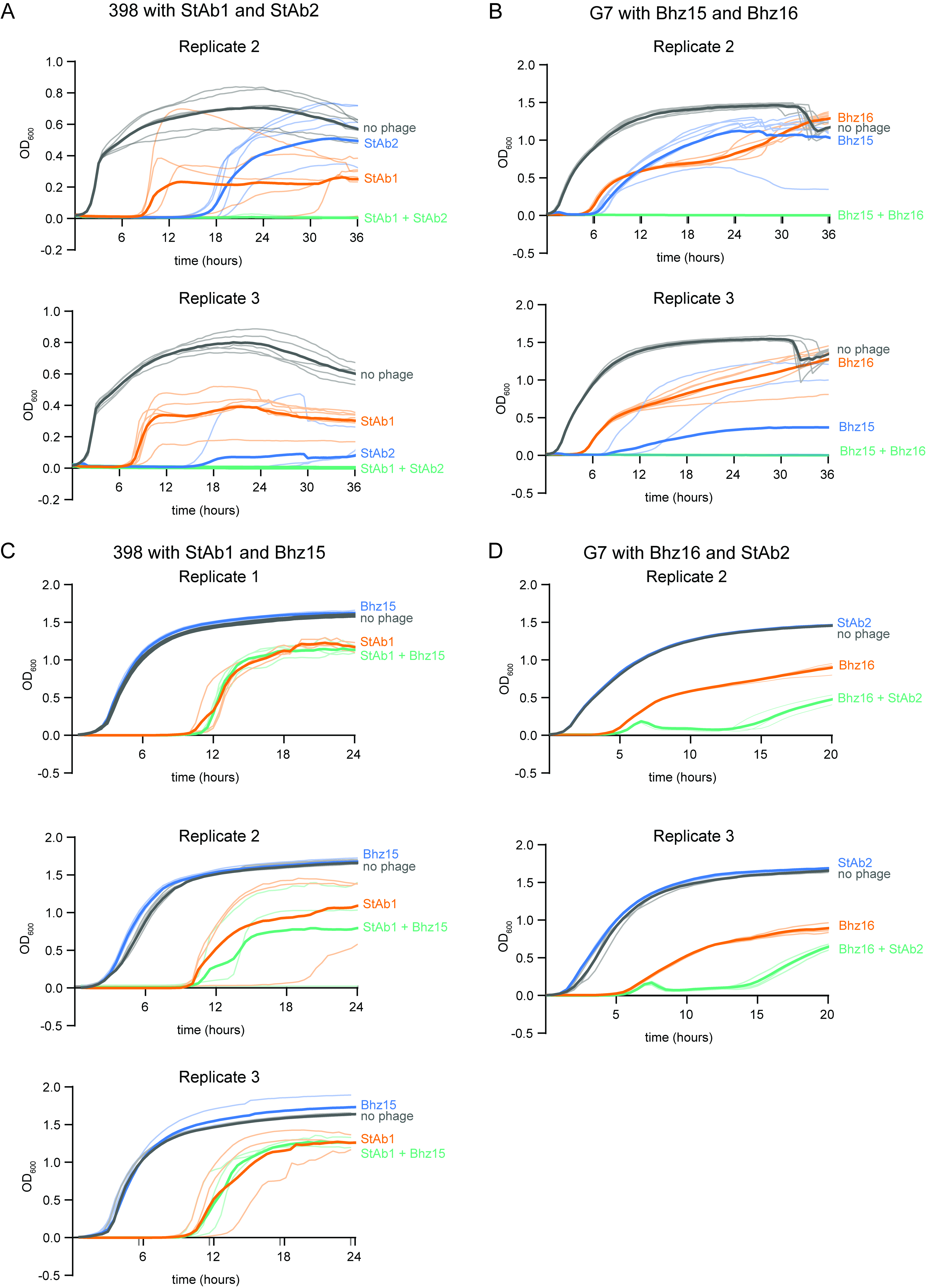

Supplement: S9 Fig — Growth of 398 (A and C) or G7 (B and D) with the indicated individual phage or phages combined. Phages were added to an MOI of 1. Each graph represents a single independent replicate with the average of three (A-B) or six (C-D) technical replicates presented as a bold line, with each technical replicate shown as a narrow line. (TIF) [file ppat.1013536.s009.tif]

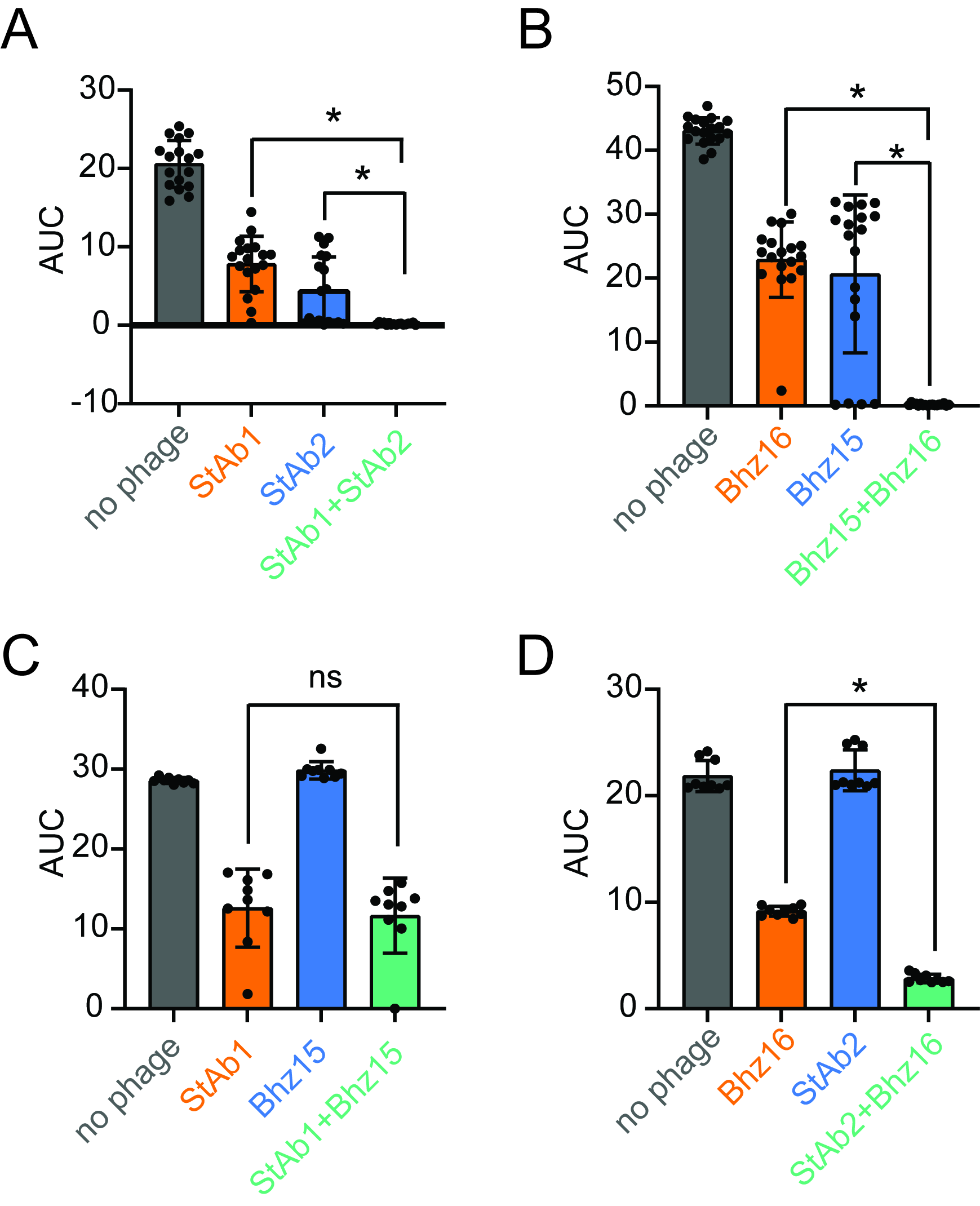

Supplement: S10 Fig — Area under the curve (AUC) calculated for growth curves of 398 (A, C) or G7 (B, D) with the indicated individual phage or phages combined from Figs 7 and S9. Each graph represents three independent replicates each with three (C-D) or six (A-B) technical replicates, with each point representing a single technical replicate. (A) corresponds to Figs 7C and S9A, (B) corresponds to Figs 7D and S9B, (C) corresponds to S9C Fig, and (D) corresponds to Figs 7E and S9D. The average is presented, error bars representing standard deviation (sd). Statistical significance was determined by a Brown-Forsythe and Welch’s ANOVA, with treatments compared using Dunnett’s T3 multiple comparisons test, * P < 0.05, ns P > 0.05. (TIF) [file ppat.1013536.s010.tif]
