# Supplementary material for: Acinetobacter phages use distinct strategies to breach the capsule barrier
Source: PLoS Pathog. 2025 Sep 29;21(9):e1013536. doi: 10.1371/journal.ppat.1013536 (PMC12507263; doi:10.1371/journal.ppat.1013536)
Supplement: S1 Table — Location and type of SNPs identified in spontaneous capsule mutants. Predicted insertions or rearrangements are reported as I/R. (PDF) [file ppat.1013536.s011.pdf]

Table S1. Capsule mutant confirmation sequencing analysis

Location and type of SNPs identified in spontaneous capsule mutants. Predicted insertions or rearrangements are reported as I/R.

Strain	Base position	Locus tag	Mutation	Protein effect	Gene name
**Ab5075-cm**	2,103,243-2,103,251	OSV63_10415	I/R		*gtr1*
**AbCAN2-cm**	3,625,987-3625988	F9K57_17390	201_202delAT	Frameshift	*wzy*
**Ab014-cm**	186,223	ACQUGU_01620	1942T>A	S648T	
	3,430,248	ACQUGU_16985	886delG	Frameshift	*gne1*
