# Supplementary material for: Acinetobacter phages use distinct strategies to breach the capsule barrier
Source: PLoS Pathog. 2025 Sep 29;21(9):e1013536. doi: 10.1371/journal.ppat.1013536 (PMC12507263; doi:10.1371/journal.ppat.1013536)
Supplement: S2 Table — Location and type of SNPs identified in phage-resistant mutants. Predicted insertions or rearrangements are reported as I/R. (PDF) [file ppat.1013536.s012.pdf]

Table S2: Phage-resistant mutant sequencing analysis

Location and type of SNPs identified in phage-resistant mutants. Predicted insertions or rearrangements are reported as I/R.

Strain	Base position	Locus tag	Mutation	Protein effect	Gene
**398-StAb1eA**	3,685,755-3,685,758	GAB398_03413	502_505 delGTAT	Frameshift	*gtr50*
**398-StAb1eB**	3,684,949	GAB398_03412	170G>A	S57L	*itrA3*
**398-StAb1eC**	1,164,283	GAB398_01081	169delT	Frameshift	*rsmE2*
	3,687,268	GAB398_03414	85G>A	Truncation	*gtr49*
**398-StAb1eD**	3,684,701	GAB398_03412	418G>A	Truncation	*itrA3*
**398-StAb1eE**	3,684,724	GAB398_03412	395G>A	T132I	*itrA3*
**398-StAb1eF**	3,685,790-3,685,791	GAB398_03413	470_471 insA	Frameshift	*gtr50*
**398-StAb1eG**	3,680,173	GAB398_03408	393delC	Frameshift	*gne1*
**398-StAb1eH**	3,295,607	GAB398_03054	644C>T	C215Y	*pcrA*
	3,680,128	GAB398_03408	438delT	Frameshift	*gne1*
**398-StAb2eA**	3,699,472	GAB398_03425	1631C>T	P544L	*wzc*
**398-StAb2eB**	2,478,252	GAB398_02309	169G>A	H57Y	*kdsD*
**398-StAb2eC**	553,993	GAB398_00538	696delT	Frameshift	A1S_2903
**398-StAb2eD**	2,477,066	GAB398_02308	374delA	Frameshift	*kdsC*
**398-StAb2eE**	2,120,115	GAB398_01979	1480G>A	R494C	*tktA*
**398-StAb2eF**	553,935-553,936	GAB398_00538	639_640 insGA	Frameshift	A1S_2903
**398-StAb2eG**	554,133	GAB398_00538	836delC	Frameshift	A1S_2903
	3,174,852	GAB398_02942	1248G>A	Synonymous	*silP*
**398-StAb2eH**	186,642	GAB398_00171	409C>T	Substitution L137F	*waaA*
**G7-Bhz16eA**	1,088,215	DFOAMG_05305	206delT	Frameshift	uspA
	2,324,820-2,324,828	DFOAMG_11475<I> DFOAMG_11480	I/R		*ata/lon*
	1,749,939-1,749,947	DFOAMG_08565>I> DFOAMG_08570	I/R		*roxA/acrR*
	3,362,113-3,362,120	DFOAMG_16365	I/R (12-19)		*fnlA*
**G7-Bhz16eB**	1,088,215	DFOAMG_05305	206delT	Frameshift	*uspA*
	1,749,939-1,749,947	DFOAMG_08565>I> DFOAMG_08570	I/R		*roxA/acrR*
	3,362,113-3,362,120	DFOAMG_16365	I/R I/R (12-19)		*fnlA*
**G7-Bhz16eC**	1,088,215	DFOAMG_05305	206delT	Frameshift	*uspA*
	1,749,939-1,749,947	DFOAMG_08565>I> DFOAMG_08570	I/R		*roxA/acrR*
	3,113,082-3,113,090	DFOAMG_15180	I/R (117-109)		*hns*
	3,362,113-3,362,120	DFOAMG_16365	I/R		*fnlA*
**UPAB1 Δ*wzy carO*::tn**	551,537-551,538	D1G37_RS02455	I/R (417-418)		*carO*
**17978 Δ*pglC*-StAb2eA**	3,541,903	ACX60_RS16680	21A>T	Synonymous	*nusG*
	1,034,601	ACX60_RS04875	110T>A	Truncation	*carO*
**17978 Δ*pglC*-StAb2eB**	1,035,129	ACX60_RS04875	638delC	Frameshift	*carO*
	3,513,830	ACX60_RS16565	1780delT	Frameshift	
	3,541,903	ACX60_RS16680	21A>T	Synonymous	*nusG*
**17978 Δ*pglC*-StAb2eC**	1,035,129	ACX60_RS04875	638delC	Frameshift	*carO*
	3,513,830	ACX60_RS16565	1780delT	Frameshift	
	3,541,903	ACX60_RS16680	21A>T	Synonymous	*nusG*
**17978 Δ*pglC*-StAb2eD**	3,541,903	ACX60_RS16680	21A>T	Synonymous	*nusG*
	1,034,601	ACX60_RS04875	110T>A	Truncation	*carO*
**17978 Δ*pglC*-StAb2eE**	1,035,129	ACX60_RS04875	638delC	Frameshift	*carO*
	3,513,830	ACX60_RS16565	1780delT	Frameshift	

	3,541,903	ACX60_RS16680	21A>T	Synonymous	*nusG*
**17978 Δ*pglC*-StAb2eF**	1,035,129	ACX60_RS04875	638delC	Frameshift	*carO*
	3,513,830	ACX60_RS16565	1780delT	Frameshift	
	3,541,903	ACX60_RS16680	21A>T	Synonymous	*nusG*
**17978 Δ*pglC*-StAb2eG**	3,541,903	ACX60_RS16680	21A>T	Synonymous	*nusG*
	1,034,601	ACX60_RS04875	110T>A	Truncation	*carO*
**17978 Δ*pglC*-StAb2eH**	3,541,903	ACX60_RS16680	21A>T	Synonymous	*nusG*
	1,034,601	ACX60_RS04875	110T>A	Truncation	*carO*
**MC47.2-StAb3eA**	2,185,299-2,185,307	ABUS04_RS10855	I/R (410-418)		*pgrD*
**MC47.2-StAb3eB**	2,185,299-2,185,307	ABUS04_RS10855	I/R (410-418)		*pgrD*
**MC47.2-StAb3eC**	2,185,299-2,185,307	ABUS04_RS10855	I/R (410-418)		*pgrD*
**MC47.2-StAb3eD**	2,185,299-2,185,307	ABUS04_RS10855	I/R (410-418)		*pgrD*
	1,663,876-1,668,323	ABUS04_RS08350-AbUS04_RS08375	Deletion		
**Up280-StAb3eA**	1,025,186	ABUS10_RS04760	419A>C	Q140P	*pgrF*
