# Supplementary material for: Acinetobacter phages use distinct strategies to breach the capsule barrier
Source: PLoS Pathog. 2025 Sep 29;21(9):e1013536. doi: 10.1371/journal.ppat.1013536 (PMC12507263; doi:10.1371/journal.ppat.1013536)
Supplement: S3 Table — K-locus and capsular polysaccharide types and structure of A. baumannii strains determined using Kaptive [24]. (PDF) [file ppat.1013536.s013.pdf]

Table S3. Capsule type of wild-type *A. baumannii* strains
K-locus and capsular polysaccharide types and structure of *A. baumannii* strains determined using Kaptive[1].

Strain	K-locus type	Capsular polysaccharide (CPS) type	Capsule structure [2]
398	KL24	K24-Wzy-GI2	β-D-Fuc*p*3NAc-<br>(1<br>↓<br>3)<br>[3)-α-D-Gal*p*-(1→6)-α-D-Glc*p*NAc-(1→4)-α-D-Gal*p*NAc-(1→3)-β-D-Glc*p*NAc-(1→][3]
MC47 LCP-	KL9	K9	α-L-Fuc*p*NAc-<br>(1<br>↓<br>4)<br>[3)-α-D-Gal*p*NAcA-(1→3)-α-L-Fuc*p*NAc-(1→3)-β-D-Glc*p*NAc-(1→] [4]
G7	KL9	K9	α-L-Fuc*p*NAc-<br>(1<br>↓<br>4)<br>[3)-α-D-Gal*p*NAcA-(1→3)-α-L-Fuc*p*NAc-(1→3)-β-D-Glc*p*NAc-(1→] [4]
Ab5075	KL25	K25	[3)β-D-Man*p*NAcA-(1→4)-β-D-Man*p*NAcA-(1→3)-α-D-Qui*p*NAc4NR-(1→] [5]
17978	KL3	K3	β-GlcNAc3NAcA4OAc-<br>(1<br>↓<br>4)<br>[3)-α-Gal*p*-(1→6)-β-Glc*p*-(1→3)-β-Gal*p*NAc-(1→]<br>(6<br>↓<br>4)<br>β-Glc*p*NAc [6]
UPAB1	KL149	unknown (likely K9[1,4])	Likely<br>α-L-Fuc*p*NAc-<br>(1<br>↓<br>4)<br>[3)-α-D-Gal*p*NAcA-(1→3)-α-L-Fuc*p*NAc-(1→3)-β-D-Glc*p*NAc-(1→] [4]
AbCAN2	KL140	unknown	Unknown
Ab014	KL120	unknown	Unknown

References

1. Cahill SM, Hall RM, Kenyon JJ. An update to the database for Acinetobacter baumannii capsular polysaccharide locus typing extends the extensive and diverse repertoire of genes found at and outside the K locus. Microbial Genomics. 2022;8(10):000878.

2. Indian Institute of Technology Hyderabad, India. K antigen structure [Internet]. ABSD:Acinetobacter baumannii K-antigen three-dimensional Structure Database. [cited 2025 Aug 12]. Available from: https://project.iith.ac.in/ABSD/k_antigen.html

3. Kenyon JJ, Kasimova AA, Shneider MM, Shashkov AS, Arbatsky NP, Popova AV, et al. The KL24 gene cluster and a genomic island encoding a Wzy polymerase contribute genes needed for synthesis of the K24 capsular polysaccharide by the multiply antibiotic resistant Acinetobacter baumannii isolate RCH51. Microbiology. 2017;163(3):355–63.

4. Kasimova AA, Sharar NS, Ambrose SJ, Knirel YA, Shneider MM, Timoshina OY, et al. The Acinetobacter baumannii K70 and K9 capsular polysaccharides consist of related K-units linked by the same Wzy polymerase and cleaved by the same phage depolymerases. Microbiol Spectr. 2023 Dec 12;11(6):e0302523.

5. Senchenkova SN, Shashkov AS, Popova AV, Shneider MM, Arbatsky NP, Miroshnikov KA, et al. Structure elucidation of the capsular polysaccharide of Acinetobacter baumannii AB5075 having the KL25 capsule biosynthesis locus. Carbohydr Res. 2015 May 18;408:8–11.

6. Lees-Miller RG, Iwashkiw JA, Scott NE, Seper A, Vinogradov E, Schild S, et al. A common pathway for -linked protein-glycosylation and synthesis of capsule in cinetobacter baumannii. Molecular Microbiology. 2013;89(5):816–30.
