# Supplementary material for: Acinetobacter phages use distinct strategies to breach the capsule barrier
Source: PLoS Pathog. 2025 Sep 29;21(9):e1013536. doi: 10.1371/journal.ppat.1013536 (PMC12507263; doi:10.1371/journal.ppat.1013536)
Supplement: S4 Table — Susceptibility of CarO mutants to imipenem based upon minimum inhibitory concentration (MIC). (PDF) [file ppat.1013536.s014.pdf]

Table S4. Imipenem MIC

Susceptibility of CarO mutants to imipenem based upon minimum inhibitory concentration (MIC).

**Strain**	**MIC (µg/mL)**			**Final MIC (µg/mL)**
	**Trial 1**	**Trial 2**	**Trial 3**	
**398**	0.25	0.25	0.25	**0.25**
**398 Δ*carO***	0.25	0.25	0.25	**0.25**
**398-StAb1eA**	0.125	0.125	0.125	**0.125**
**398-StAb1eA Δ*carO***	0.125	0.125	0.125	**0.125**
**17978 Δ*pglC***	0.25	0.25	0.25	**0.25**
**17978 Δ*pglC*-StAb2eA**	0.5	0.25	0.5	**0.25-0.5**
**UPAB1 Δ*wzy***	64	32	32	**32-64**
**UPAB1 Δ*wzy* Tn::*carO***	32	32	32	**32**
