# Supplementary material for: Acinetobacter phages use distinct strategies to breach the capsule barrier
Source: PLoS Pathog. 2025 Sep 29;21(9):e1013536. doi: 10.1371/journal.ppat.1013536 (PMC12507263; doi:10.1371/journal.ppat.1013536)
Supplement: S5 Table — Structural alignment of a subset of exopolysaccharide synthesis and transport proteins described in Fig 5C-D using RCSB PDB pairwise structure alignment [105]. (PDF) [file ppat.1013536.s015.pdf]

Table S5. Structural alignment of exopolysaccharide synthesis and transport proteins.

Structural alignment of a subset of exopolysaccharide synthesis and transport proteins described in Figure 5C-D using RCSB PDB pairwise structure alignment[1].

Aligned to	Protein	ID	RMSD	TM-Score	Identity	Aligned residues	Sequence length	Modeled residues
A. baumannii **PgrA D0CBT8**	*E. coli* DgcJ	P76237	3.44	0.39	8%	102	496	496
	E. coli DgcQ	P76330	3.31	0.4	15%	106	564	564
A. baumannii **PgrC D0CBT6**	*E. coli* BcsA	P37653	3.09	0.82	17%	347	872	872
	A. baumannii PgaC	C8YYH7	2.78	0.83	23%	348	392	392
	E. coli NfrB	P0AFA5	3.19	0.86	17%	363	745	745
	P. soli NfrB	A0A1H9LH73	3.22	0.86	16%	359	724	724
A. baumannii **PgrD D0CBT5**	*E. coli* BcsB	P37652	4.24	0.75	9%	446	779	779
A. baumannii **PgrE D0CBT4**	*A. baumannii* PgaB	C8YYH6	4.05	0.63	13%	197	510	510
	E. coli YbcH	P37325	3.28	0.65	21%	199	296	296
A. baumannii **PgrF D0CBT3**	*E. coli* WecB	P27828	1.64	0.94	42%	371	376	376
A. baumannii **PgrG D0CBT2**	*E. coli* BcsC	P37650	6.05	0.53	8%	157	1157	1157
	A. baumannii PgaA	C8YYH5	6.34	0.48	6%	155	747	747
	E. coli NfrA	P31600	4.25	0.67	11%	258	990	990

References

1. Bittrich S, Segura J, Duarte JM, Burley SK, Rose Y. RCSB protein Data Bank: exploring protein 3D similarities via comprehensive structural alignments. Bioinformatics. 2024 Jun 3;40(6):btae370.
