# Supplementary material for: Acinetobacter phages use distinct strategies to breach the capsule barrier
Source: PLoS Pathog. 2025 Sep 29;21(9):e1013536. doi: 10.1371/journal.ppat.1013536 (PMC12507263; doi:10.1371/journal.ppat.1013536)
Supplement: S6 Table — All strains with published Mystique or EAb13 host range, or strains for which we measured StAb3 host range, were analyzed for the presence and status of the PGR locus. Summary data is provided at the bottom of the table. See also Fig 6A for a graphical summary [32,33]. (PDF) [file ppat.1013536.s016.pdf]

Table S6: Description of PGR locus present in strains tested for StAb3-like phage susceptibility.

All strains with published Mystique or EAb13 host range, or strains for which we measured StAb3 host range, were analyzed for the presence and status of the PGR locus. Summary data is provided at the bottom of the table. See also Figure 6A for a graphical summary.

Strain	PGR genotype	StAb3 infection	Mystique infection	EAb13 infection
MRSN334	Normal	n/a	N*	Y
MRSN843	Normal	n/a	Y	Y
MRSN918	Normal	n/a	Y	Y
MRSN959	Normal	n/a	Y	Y
MRSN960	Normal	n/a	Y	Y
MRSN1171	Normal	n/a	N*	Y
MRSN1174	Normal	n/a	Y	Y
MRSN1183	Normal	n/a	Y	Y
MRSN1187	Normal	n/a	Y	Y
MRSN1196	Normal	n/a	Y	Y
MRSN1311	Normal	n/a	Y	Y
MRSN1551	Normal	n/a	Y	Y
MRSN2821	Not found	n/a	N	N
MRSN3360	Not found	n/a	N	N
MRSN3658	Normal	n/a	Y	Y
MRSN3692	Normal	n/a	Y	Y
MRSN3874	Normal	n/a	Y	Y
MRSN4484	Normal	n/a	Y	Y
MRSN4943	Normal	n/a	Y	Y
MRSN5969	Pseudogene (*pgrB*)	n/a	N	N
MRSN6541	Normal	n/a	Y	Y
MRSN7067	Normal	n/a	Y	N
MRSN7113	Normal	n/a	Y	Y
MRSN7124	Normal	n/a	Y	Y
MRSN7137	Normal	n/a	Y	Y
MRSN7153	Normal	n/a	N*	Y
MRSN7213	Normal	n/a	Y	Y
MRSN7251	Normal	n/a	Y	Y
MRSN7431	Normal	n/a	Y	Y
MRSN7446	Normal	n/a	Y	Y
MRSN7460	Normal	n/a	Y	Y
MRSN7521	Normal	n/a	Y	Y
MRSN7576	Normal	n/a	Y	Y
MRSN7690	Normal	n/a	Y	Y
MRSN7725	Normal	n/a	Y	Y
MRSN7735	Normal	n/a	Y	Y
MRSN10372	Normal	n/a	Y	Y

MRSN11224	Normal	n/a	Y	Y
MRSN11663	Normal	n/a	Y	Y
MRSN11669	Normal	n/a	Y	Y
MRSN11695	Normal	n/a	Y	Y
MRSN11703	Normal	n/a	Y	Y
MRSN11816	Pseudogene (*pgrC*)	n/a	N*	N
MRSN14193	Normal	n/a	Y	Y
MRSN14237	Normal	n/a	Y	Y
MRSN14427	Normal	n/a	Y	Y
MRSN15049	Normal	n/a	Y	Y
MRSN15070	Normal	n/a	Y	Y
MRSN15075	Normal	n/a	Y	Y
MRSN15088	Normal	n/a	Y	Y
MRSN15093	Normal	n/a	Y	Y
MRSN15129	Normal	n/a	Y	Y
MRSN15574	Normal	n/a	Y	Y
MRSN16880	Normal	n/a	Y	Y
MRSN17493	Normal	n/a	Y	Y
MRSN19482	Normal	n/a	Y	Y
MRSN21660	Normal	n/a	Y	Y
MRSN21681	Unclear, split between multiple contigs	n/a	N	N
MRSN22112	Two intact genes (*pgrA*, *pgrB*) and two pseudogenes (*pgrC*, *pgrG*)	n/a	N*	N
MRSN23390	Normal	n/a	Y	Y
MRSN24008	Normal	n/a	Y	Y
MRSN24603	Pseudogene (*pgrB*)	n/a	Y	N
MRSN25547	Normal	n/a	Y	Y
MRSN29908	Normal	n/a	Y	Y
MRSN29999	Normal	n/a	Y	Y
MRSN30000	Normal	n/a	Y	Y
MRSN30885	Normal	n/a	Y	Y
MRSN30896	Normal	n/a	Y	Y
MRSN30909	Normal	n/a	Y	Y
MRSN30912	Normal	n/a	Y	Y
MRSN30945	Pseudogene (*pgrF*)	n/a	N	N
MRSN31159	Normal	n/a	Y	Y
MRSN31196	Normal	n/a	Y	Y
MRSN31461	Normal	n/a	Y	Y
MRSN31468	Normal	n/a	Y	Y
MRSN31523	Normal	n/a	Y	Y
MRSN31915	Normal	n/a	Y	Y
MRSN31937	Unclear, split between multiple contigs	n/a	N	N

MRSN31942	Normal	n/a	Y	Y
MRSN31947	Normal	n/a	Y	Y
MRSN32076	Pseudogene (*pgrC*)	n/a	N	N
MRSN32104	Normal	n/a	Y	Y
MRSN32108	Normal	n/a	Y	Y
MRSN32142	Normal	n/a	Y	Y
MRSN32304	Unclear, at the end of a contig	n/a	N	N
MRSN32797	Normal	n/a	Y	Y
MRSN32842	Normal	n/a	Y	Y
MRSN32865	Normal	n/a	Y	Y
MRSN32866	Normal	n/a	Y	Y
MRSN32875	Normal	n/a	Y	Y
MRSN32892	Normal	n/a	Y	Y
MRSN32915	Normal	n/a	Y	Y
MRSN337038	Unclear, split between multiple contigs	n/a	N*	N
MRSN351162	Normal	n/a	Y	Y
MRSN351524	Normal	n/a	Y	Y
MRSN423159	Normal	n/a	Y	Y
MRSN480561	Normal	n/a	Y	Y
MRSN480622	Normal	n/a	N	N
MRSN489669	Normal	n/a	Y	Y
MRSN489678	Normal	n/a	Y	Y
17978	Pseudogene (*pgrF*)	Y	n/a	n/a
Ab014	Normal	Y	n/a	n/a
AB5075	Normal	Y	n/a	n/a
AbCAN2	Normal	Y	n/a	n/a
G7	Normal	Y	n/a	n/a
Hv373	Normal	Y	n/a	n/a
Hv383	Normal	Y	n/a	n/a
Hv431	Not found	N	n/a	n/a
Hv455	Normal	Y	n/a	n/a
Hv635	Not found	N	n/a	n/a
Hv637	Normal	Y	n/a	n/a
Hv640	Not found	N	n/a	n/a
Hv652	Normal	Y	n/a	n/a
Hv766	Normal	Y	n/a	n/a
Hv770	Normal	Y	n/a	n/a
Hv780	Not found	N	n/a	n/a
Hv781	Not found	N	n/a	n/a
Hv782	Not found	N	n/a	n/a
HvAb04	Normal	Y	n/a	n/a
Lv347	Normal	Y	n/a	n/a

Lv371	Normal	Y	n/a	n/a
Lv419	Normal	Y	n/a	n/a
Lv643	Normal	Y	n/a	n/a
Lv646	Normal	Y	n/a	n/a
Lv647	Normal	Y	n/a	n/a
Lv648	Normal	Y	n/a	n/a
MC47	Normal	Y	n/a	n/a
Rp376	Normal	Y	n/a	n/a
Rp426	Normal	Y	n/a	n/a
Rp428	Normal	Y	n/a	n/a
Rp436	Normal	Y	n/a	n/a
Rp654	Normal	Y	n/a	n/a
Rp668	Normal	Y	n/a	n/a
Rp772	Normal	Y	n/a	n/a
Rp779	Pseudogene (*pgrG*) with ISAba22 family transposase inserted	N	n/a	n/a
Up280	Normal	Y	n/a	n/a
Up367	Normal	Y	n/a	n/a
Up377	Normal	Y	n/a	n/a
Up392	Normal	Y	n/a	n/a
Up398	Normal	Y	n/a	n/a
Up423	Normal	Y	n/a	n/a
Up425	Not found	N	n/a	n/a
Up438	Normal	Y	n/a	n/a
Up644	Normal	Y	n/a	n/a
Up645	Normal	Y	n/a	n/a
UPAB1	Pseudogenes (*pgrD*, *pgrE*)	Y	n/a	n/a
ADP1	Normal	Y	Y	n/a
M2	Normal	Y	Y	n/a
**Summary data**				
**EAb13**	Can infect	Cannot infect	Fisher's exact test:	
PGR locus intact	86	2	$p < 0.0001$	
PGR locus not intact	0	12		
**Mystique**	Can infect	Cannot infect	Fisher's exact test:	
PGR locus intact	84	4	$p < 0.0001$	
PGR locus not intact	1	11		
**StAb3**	Can infect	Cannot infect	Fisher's exact test:	
PGR locus intact	36	0	$p < 0.0001$	
PGR locus not intact	2	8		
