# Supplementary material for: Acinetobacter phages use distinct strategies to breach the capsule barrier
Source: PLoS Pathog. 2025 Sep 29;21(9):e1013536. doi: 10.1371/journal.ppat.1013536 (PMC12507263; doi:10.1371/journal.ppat.1013536)
Supplement: S7 Table — List of bacterial strains used in the paper. (PDF) [file ppat.1013536.s017.pdf]

Table S7. Bacterial Strains

List of bacterial strains used in the paper.

***Acinetobacter baumannii* strains**

Bacterial Strain	Description	Source	Genome assembly	Biosample	ID
**398**	*A. baumannii* isolated from a urinary tract infection in 2019 from IHMA Labs, also referred to as Up398	[1]	CP191189		D747
**MC47**	*A. baumannii* isolated from a tracheal secretion in 2016 in Cochabamba, Bolivia, also called Rp342	[2]	GCA_051776275.1	SAMN48318281	D765
**MC47.2**	MC47 that has been cured of its LCP	This study		SAMN48318239	D766
**G7**	*A. baumannii* isolated from patient sputum in Georgia- DSM25645	DSMZ		SAMN48318240	D732
**Ab5075**	*A. baumannii* isolated from a tibia with osteomyelitis	[3]	GCA_028583505.1	SAMN48318241	D750
**17978**	ATCC 17978	[4]	GCA_001077675.1		B83
**UPAB1**	*A. baumannii* isolated from a urinary tract infection in 2016 in Buenos Aires, Argentina	[5,6]	GCA_006843645.1		B539
**AbCAN2**	*A. baumannii* isolated from a coccyx (formerly Ab1225)	[7,8]	GCA_009833005.1	SAMN48318242	D754
**Ab014**	*A. baumannii* isolated from a patient in an ICU in Montreal, QC, Canada	[9]	CP191187-CP191188	SAMN48318243	D752
**17978 Δ*pglC***	Capsule mutant of 17978 with *pglC* (A1S_0061) deletion	[10]		SAMN48318244	D749
**17978 Δ*pglC*:PRLM2**	Complementation of 17978 Δ*pglC* with *pglC* on plasmid PRLM2	[10]			A670
**17978 Δ*pglC*:pWH1266**	Empty vector control for 17978 Δ*pglC* PRLM2	[10]			A671
**Ab5075-cm**	Spontaneous capsule mutant of Ab5075 *gtr1*::ISAba13	This study		SAMN48318245	D751
**UPAB1 Δ*wzy***	Capsule mutant of UPAB1 with *wzy* deletion	This study		SAMN50643694	D748
**AbCAN2-cm**	Spontaneous capsule mutant of AbCAN2 *wzy* (A)5->6	This study		SAMN48318246	D755
**Ab014-cm**	Spontaneous capsule mutant of Ab014 *wzy* Δbp1	This study		SAMN48318247	D753
**398-StAb1eA**	398 StAb1 escape mutant from planktonic culture	This study		SAMN48318248	D772
**398-StAb1eB**	398 StAb1 escape mutant from planktonic culture	This study		SAMN48318249	D773
**398-StAb1eC**	398 StAb1 escape mutant from planktonic culture	This study		SAMN48318250	D774
**398-StAb1eD**	398 StAb1 escape mutant from planktonic culture	This study		SAMN48318251	D775
**398-StAb1eE**	398 StAb1 escape mutant from planktonic culture	This study		SAMN48318252	D776
**398-StAb1eF**	398 StAb1 escape mutant from planktonic culture	This study		SAMN48318253	D777
**398-StAb1eG**	398 StAb1 escape mutant from planktonic culture	This study		SAMN48318254	D778
**398-StAb1eH**	398 StAb1 escape mutant from planktonic culture	This study		SAMN48318255	D779

**G7-Bhz16eA**	G7 Bhz16 escape mutant from liquid culture	This study		SAMN48318256	D797
**G7-Bhz16eB**	G7 Bhz16 escape mutant from liquid culture	This study		SAMN48318257	D798
**G7-Bhz16eC**	G7 Bhz16 escape mutant from liquid culture	This study		SAMN48318258	D799
**398-StAb2eA**	398 StAb2 escape mutant from solid media	This study		SAMN48318259	D764
**398-StAb2eB**	398 StAb2 escape mutant from planktonic culture	This study		SAMN48318260	D782
**398-StAb2eC**	398 StAb2 escape mutant from planktonic culture	This study		SAMN48318261	D783
**398-StAb2eD**	398 StAb2 escape mutant from planktonic culture	This study		SAMN48318262	D784
**398-StAb2eE**	398 StAb2 escape mutant from planktonic culture	This study		SAMN48318263	D785
**398-StAb2eF**	398 StAb2 escape mutant from planktonic culture	This study		SAMN48318264	D786
**398-StAb2eG**	398 StAb2 escape mutant from planktonic culture	This study		SAMN48318265	D787
**398-StAb2eH**	398 StAb2 escape mutant from planktonic culture	This study		SAMN48318266	D788
**398 Δ*lpsB***	398 with *lpsB* deletion	[11]			D770
**398 Δ*lpsB*+*lpsB***	398 Δ*lpsB* with *lpsB* complementation	[11]			D769
**UPAB1 Δ*wzy carO*::tn**	UPAB1 Δ*wzy* with transposon inserted in *carO* from pNJW684 resistant to StAb2	This study		SAMN48318267	D346
**17978 Δ*pglC*-StAb2eA**	17978 Δ*pglC* StAb2 escape mutant from liquid culture	This study		SAMN48318268	D756
**17978 Δ*pglC*-StAb2eB**	17978 Δ*pglC* StAb2 escape mutant from liquid culture	This study		SAMN48318269	D757
**17978 Δ*pglC*-StAb2eC**	17978 Δ*pglC* StAb2 escape mutant from liquid culture	This study		SAMN48318270	D758
**17978 Δ*pglC*-StAb2eD**	17978 Δ*pglC* StAb2 escape mutant from liquid culture	This study		SAMN48318271	D759
**17978 Δ*pglC*-StAb2eE**	17978 Δ*pglC* StAb2 escape mutant from liquid culture	This study		SAMN48318272	D760
**17978 Δ*pglC*-StAb2eF**	17978 Δ*pglC* StAb2 escape mutant from liquid culture	This study		SAMN48318273	D761
**17978 Δ*pglC*-StAb2eG**	17978 Δ*pglC* StAb2 escape mutant from liquid culture	This study		SAMN48318274	D762
**17978 Δ*pglC*-StAb2eH**	17978 Δ*pglC* StAb2 escape mutant from liquid culture	This study		SAMN48318275	D763
**ACICU**	*A. baumannii* isolate from an outbreak in Rome, Italy	[12]	GCA_000018445.1		B916
**AYE**	*A. baumannii* isolate from human blood in 2003	[13]	GCA_000069245.1		D200
**19606**	A. *baumannii* model strain recovered from a urine sample	[14]	GCA_009035845.1		A695
**UPAB1 Δ*wzy carO*::tn + *carO*^{19606}**	UPAB1 Δ*wzy carO*::tn with the *carO* gene from 19606 inserted at the *att*Tn7 site by pUC18T-miniTn7-Zeo	This study			D793
**UPAB1 Δ*wzy carO*::tn + *carO*^{398}**	UPAB1 Δ*wzy carO*::tn with the *carO* gene from 398 inserted at the *att*Tn7 site by pUC18T-miniTn7-Zeo	This study			D795
**UPAB1 Δ*wzy carO*::tn + *carO*^{AYE}**	UPAB1 Δ*wzy carO*::tn with the *carO* gene from AYE inserted at the *att*Tn7 site by pUC18T-miniTn7-Zeo	This study			D796

**UPAB1 Δ*wzy carO*::tn + $carO^{ACICU}$**	UPAB1 Δ*wzy carO*::tn with the *carO* gene from ACICU inserted at the *att*Tn7 site by pUC18T-miniTn7-Zeo	This study			D794
**398 Δ*carO***	398 with *carO* deletion	This study			D780
**398-StAb1eA Δ*carO***	398-StAb1E1 with c*arO* deletion	This study			D781
**MC47.2-StAb3eA**	MC47.2 StAb3 escape mutant from an LB-agar plate incubated at 30°C	This study		SAMN48318276	D800
**MC47.2-StAb3eB**	MC47.2 StAb3 escape mutant from an LB-agar plate incubated at 30°C	This study		SAMN48318277	D801
**MC47.2-StAb3eC**	MC47.2 StAb3 escape mutant from an LB-agar plate incubated at 20°C	This study		SAMN48318278	D802
**MC47.2-StAb3eD**	MC47.2 StAb3 escape mutant from an LB-agar plate with chloramphenicol incubated at 30°C	This study		SAMN48318279	D803
**Up280**	*A. baumannii* strain isolated from a catheter	[15]	GCA_04215 2325.1		D767
**Up280-StAb3eA**	Up280 StAb3 escape mutant from an LB-agar plate incubated at RT	This study		SAMN48318280	D768
**MC47.2-StAb3eA + *pgrD***	MC47.2-StAb3eA with the *pgrF* gene from MC47.2 inserted at the *att*Tn7 site by pUC18T-miniTn7-Apr	This study			E015
**Up280-StAb3eA + *pgrF***	Up280-StAb3eA with the *pgrF* gene from MC47.2 inserted at the *att*Tn7 site by pUC18T-miniTn7-Apr	This study			E016
**MC47.2 Δ*pgrD***	MC47.2 with *pgrD* deletion	This study			E017
**MC47.2 Δ*pgrD* + *pgrD***	MC47.2 Δ*pgrD* with the *pgrD* gene from MC47.2 inserted at the *att*Tn7 site by pUC18T-miniTn7-Apr	This study			E018

Other *Acinetobacter* strains

**Species**	**Strain**	**Description**	**Source**	**Genome assembly**	**ID**
A. baylyi	ADP1	*A. baylyi* model organism	[16]	GCA_000046845.1	KHM50
**A. calcoaceticus**	1217	*A. calcoaceticus* clinical isolate from urine	This study		KHM51
**A. junii**	1223	*A. junii* clinical isolate from endotracheal tube	This study		KHM54
A. nosocomialis	M2	*A. nosocomialis* clinical isolate from hip infection	[17]	GCA_005281455.1	KHM53
A. nosocomialis	Up413	*A. nosocomialis* clinical isolate ACATPM 4061-2 from the University of Pittsburgh	[18]		Y413
A. pittii	1219	*A. pittii* clinical isolate from urine	This study		KHM52
A. pittii	Lv378	*A. pittii* clinical isolate from tracheal aspirate, also called 1057803	JMI Labs		Y378

***Escherichia coli* strains**

**Bacterial Strain**	**Description**	**Source**	**ID**
***E. coli* + $carO^{19606}$**	*E. coli* Stellar Competent cells expressing CarO from 19606 with pUC18T-miniTn7T-zeo- $carO^{19606}$	This study	D375
E. coli* + *pilA	*E. coli* Stellar Competent cells expressing PilA with pUC18T-miniTn7T-zeo-ARC_*pilA*	[19]	D335
**HB101 (pRK2013)**	*E. coli* carrying plasmid to facilitate conjugation in tri and four-parental mating	[20]	A510
**100D (pTNS2)**	*E. coli* carrying plasmid expressing a transposase for four-parental mating	[21]	B72
**S17-1 λpir**	*E. coli* strain with λpir, allowing for replication of pJNW684	ATCC- BAA-2428	A594
**S17-1 λpir (pJNW684-Apr)**	*E. coli* carrying plasmid pJNW684-Apr for introducing a transposon with an apramycin resistance gene	This study	D276

References

1. Distel JS, Di Venanzio G, Mackel JJ, Rosen DA, Feldman MF. Replicative Acinetobacter baumannii strains interfere with phagosomal maturation by modulating the vacuolar pH. PLoS Pathog. 2023 Jun;19(6):e1011173.

2. Cerezales M, Xanthopoulou K, Wille J, Bustamante Z, Seifert H, Gallego L, et al. Acinetobacter baumannii analysis by core genome multi-locus sequence typing in two hospitals in Bolivia: endemicity of international clone 7 isolates (CC25). International Journal of Antimicrobial Agents. 2019 Jun 1;53(6):844–9.

3. Jacobs AC, Thompson MG, Black CC, Kessler JL, Clark LP, McQueary CN, et al. AB5075, a Highly Virulent Isolate of Acinetobacter baumannii, as a Model Strain for the Evaluation of Pathogenesis and Antimicrobial Treatments. mBio. 2014 May 27;5(3):10.1128/mbio.01076-14.

4. Piechaud M, Second L. [Studies of 26 strains of Moraxella Iwoffi]. Ann Inst Pasteur (Paris). 1951 Jan;80(1):97–9.

5. Di Venanzio G, Flores-Mireles AL, Calix JJ, Haurat MF, Scott NE, Palmer LD, et al. Urinary tract colonization is enhanced by a plasmid that regulates uropathogenic Acinetobacter baumannii chromosomal genes. Nat Commun. 2019 Jun 24;10(1):2763.

6. Di Venanzio G, Moon KH, Weber BS, Lopez J, Ly PM, Potter RF, et al. Multidrug-resistant plasmids repress chromosomally encoded T6SS to enable their dissemination. Proceedings of the National Academy of Sciences. 2019 Jan 22;116(4):1378–83.

7. Weber BS, Miyata ST, Iwashkiw JA, Mortensen BL, Skaar EP, Pukatzki S, et al. Genomic and Functional Analysis of the Type VI Secretion System in Acinetobacter. PLOS ONE. 2013 Jan 24;8(1):e55142.

8. Lopez J, Ly PM, Feldman MF. The Tip of the VgrG Spike Is Essential to Functional Type VI Secretion System Assembly in Acinetobacter baumannii. mBio. 2020 Jan 14;11(1):10.1128/mbio.02761-19.

9. Fernando D, Zhanel G, Kumar A. Antibiotic Resistance and Expression Of Resistance-Nodulation-Division Pump- and Outer Membrane Porin-Encoding Genes in Acinetobacter Species Isolated from Canadian Hospitals. Canadian Journal of Infectious Diseases and Medical Microbiology. 2013;24(1):696043.

10. Lees-Miller RG, Iwashkiw JA, Scott NE, Seper A, Vinogradov E, Schild S, et al. A common pathway for -linked protein-glycosylation and synthesis of capsule in cinetobacter baumannii. Molecular Microbiology. 2013;89(5):816–30.

11. Janet-Maitre M, Venanzio GD, Jackson-Litteken CD, Scott NE, Feldman MF. Intracellular Acinetobacter baumannii Acts as a Reservoir in Lung Infection via a “Persist and Resist” Strategy [Internet]. bioRxiv; 2025 [cited 2025 Apr 29]. p. 2025.04.28.651116. Available from: https://www.biorxiv.org/content/10.1101/2025.04.28.651116v1

12. Iacono M, Villa L, Fortini D, Bordoni R, Imperi F, Bonnal RJP, et al. Whole-Genome Pyrosequencing of an Epidemic Multidrug-Resistant Acinetobacter baumannii Strain Belonging to the European Clone II Group. Antimicrob Agents Chemother. 2008 Jul;52(7):2616–25.

13. Fournier PE, Vallenet D, Barbe V, Audic S, Ogata H, Poirel L, et al. Comparative Genomics of Multidrug Resistance in Acinetobacter baumannii. PLOS Genetics. 2006 Jan 13;2(1):e7.

14. Hamidian M, Blasco L, Tillman LN, To J, Tomas M, Myers GSA. Analysis of Complete Genome Sequence of Acinetobacter baumannii Strain ATCC 19606 Reveals Novel Mobile Genetic Elements and Novel Prophage. Microorganisms. 2020 Nov 24;8(12):1851.

15. Nye TM, Zou Z, Obernuefemann CLP, Pinkner JS, Lowry E, Kleinschmidt K, et al. Microbial co-occurrences on catheters from long-term catheterized patients. Nat Commun. 2024 Jan 2;15:61.

16. Barbe V, Vallenet D, Fonknechten N, Kreimeyer A, Oztas S, Labarre L, et al. Unique features revealed by the genome sequence of Acinetobacter sp. ADP1, a versatile and naturally transformation competent bacterium. Nucleic Acids Res. 2004;32(19):5766–79.

17. Pehde B, Lizer N, Carruthers M. Complete Genome Sequence of the Nosocomial Pathogen Acinetobacter nosocomialis Strain M2. Microbiology Resource Announcements. 2019 Oct 31;8(44):10.1128/mra.00538-19.

18. Sycz G, Di Venanzio G, Distel JS, Sartorio MG, Le NH, Scott NE, et al. Modern Acinetobacter baumannii clinical isolates replicate inside spacious vacuoles and egress from macrophages. PLoS Pathog. 2021 Aug 9;17(8):e1009802.

19. Bisaro F, Jackson-Litteken CD, McGuffey JC, Hooppaw AJ, Bodrog S, Jebeli L, et al. Diclofenac sensitizes multi-drug resistant Acinetobacter baumannii to colistin. PLoS Pathog. 2024 Nov;20(11):e1012705.

20. Figurski DH, Helinski DR. Replication of an origin-containing derivative of plasmid RK2 dependent on a plasmid function provided in trans. Proceedings of the National Academy of Sciences. 1979 Apr;76(4):1648–52.

21. Choi KH, Gaynor JB, White KG, Lopez C, Bosio CM, Karkhoff-Schweizer RR, et al. A Tn7-based broad-range bacterial cloning and expression system. Nat Methods. 2005 Jun;2(6):443–8.
