# Supplementary material for: Acinetobacter phages use distinct strategies to breach the capsule barrier
Source: PLoS Pathog. 2025 Sep 29;21(9):e1013536. doi: 10.1371/journal.ppat.1013536 (PMC12507263; doi:10.1371/journal.ppat.1013536)
Supplement: S8 Table — List of all bacteriophages with their isolation and propagation hosts used in this paper. (PDF) [file ppat.1013536.s018.pdf]

Table S8: Bacteriophages

List of all bacteriophages with their isolation and propagation hosts used in this paper.

Bacteriophage	Isolation host	Propagation host	Biosample Accession number	Genbank Accession number	Source
StAb1	398	398	SAMN48412507	PX215756	This study
StAb2	398	UPAB1 Δ*wzy*	SAMN48412508	PX215757	This study
StAb3	MC47.2	MC47.2	SAMN48412509	PX215758	This study
Bhz15	G7	G7	SAMN48412510		DSMZ
Bhz16	G7	G7	SAMN48412511		DSMZ
