# Supplementary material for: Acinetobacter phages use distinct strategies to breach the capsule barrier
Source: PLoS Pathog. 2025 Sep 29;21(9):e1013536. doi: 10.1371/journal.ppat.1013536 (PMC12507263; doi:10.1371/journal.ppat.1013536)
Supplement: S9 Table — List of all plasmids used in the paper. (PDF) [file ppat.1013536.s019.pdf]

Table S9: Plasmids

List of all plasmids used in the paper.

Plasmid	Description	Source
**pKD4-Apr**	Source of FRT-flanked apramycin resistance cassette for generation of UPAB1 Δ*wzy*	[1]
**pAT04-Hygro**	Plasmid encoding RecAB recombinase for facilitating allelic exchange in generation of UPAB1 Δ*wzy*	[1–3]
**pAT03-Hygro**	Plasmid encoding IPTG-inducible copy of FLP recombinase to remove the antibiotic resistance cassette for generation of UPAB1 Δ*wzy*	[1–3]
**pJNW684**	Plasmid encoding the Himar1 mariner transposon system to generate transposon insertion mutants in *A. baumannii*	[4]
**pUC18T-miniTn7T-Apr**	Source for apramycin cassette for pJNW684-Apr	[5]
**pJNW684-Apr**	pNJW684 with the kanamycin resistance cassette exchanged for an apramycin resistance cassette	This study
**pUC18T-miniTn7T-LAC-zeo**	Vector carrying a mini-Tn7 system for insertion of genes in the *A. baumannii* chromosome downstream of the glmS gene at the attTn7 site.	[6]
**pUC18T-miniTn7T-zeo-ACICU_*carO***	pUC18T-miniTn7T-zeo with *carO* amplified from ACICU for complementation	This study
**pUC18T-miniTn7T-zeo-19606_*carO***	pUC18T-miniTn7T-zeo with the *carO* amplified from 19606 for complementation	This study
**pUC18T-miniTn7T-zeo-ARC_*pilA***	pUC18T-miniTn7T-zeo with the gene *pilA* used as a negative control for plasmid pUC18T-miniTn7T-zeo	[7]
**pEX18Ap**	Plasmid to generate mutants using allelic exchange and sucrose counter-selection	[8]
**pEX18Ap_*carO*398**	Plasmid for generation of carO deletion mutants	This study
**pEX18Ap_*pgrD***	Plasmid for generation of *pgrD* deletion mutants in MC47.2	This study
**pUC18T-miniTn7T-Apr-LAC**	Vector carrying an apramycin resistance gene and a mini-Tn7 system for insertion of genes in the *A. baumannii* chromosome downstream of the glmS gene at the attTn7 site under control of the *tac* promoter.	[6]
**pUC18T-miniTn7T-Apr-LAC-*pgrD***	pUC18T-miniTn7T-Apr-LAC with the gene *pgrD* amplified from MC47.2	This study
**pUC18T-miniTn7T-Apr-LAC-*pgrF***	pUC18T-miniTn7T-Apr-LAC with the gene *pgrF* amplified from MC47.2	This study

References

1. McGuffey JC, Jackson-Litteken CD, Di Venanzio G, Zimmer AA, Lewis JM, Distel JS, et al. The tRNA methyltransferase TrmB is critical for Acinetobacter baumannii stress responses and pulmonary infection. mBio. 2023 Aug 17;14(5):e01416-23.

2. Di Venanzio G, Flores-Mireles AL, Calix JJ, Haurat MF, Scott NE, Palmer LD, et al. Urinary tract colonization is enhanced by a plasmid that regulates uropathogenic Acinetobacter baumannii chromosomal genes. Nat Commun. 2019 Jun 24;10(1):2763.

3. Tucker AT, Powers MJ, Trent MS, Davies BW. RecET-Mediated Recombineering in Acinetobacter baumannii. In: Biswas I, Rather PN, editors. Acinetobacter baumannii: Methods and Protocols [Internet]. New York, NY: Springer; 2019 [cited 2025 Feb 23]. p. 107–13. Available from: https://doi.org/10.1007/978-1-4939-9118-1_11

4. Kazi MI, Schargel RD, Boll JM. Generating Transposon Insertion Libraries in Gram-Negative Bacteria for High-Throughput Sequencing. J Vis Exp. 2020 Jul 7;(161):10.3791/61612.

5. Jackson-Litteken CD, Di Venanzio G, Le NH, Scott NE, Djahanschiri B, Distel JS, et al. InvL, an Invasin-Like Adhesin, Is a Type II Secretion System Substrate Required for Acinetobacter baumannii Uropathogenesis. mBio. 13(3):e00258-22.

6. Ducas-Mowchun K, De Silva PM, Crisostomo L, Fernando DM, Chao TC, Pelka P, et al. Next Generation of Tn7-Based Single-Copy Insertion Elements for Use in Multi- and Pan-Drug-Resistant Strains of Acinetobacter baumannii. Applied and Environmental Microbiology. 2019 May 16;85(11):e00066-19.

7. Bisaro F, Jackson-Litteken CD, McGuffey JC, Hooppaw AJ, Bodrog S, Jebeli L, et al. Diclofenac sensitizes multi-drug resistant Acinetobacter baumannii to colistin. PLoS Pathog. 2024 Nov;20(11):e1012705.

8. Jackson-Litteken CD, Venanzio GD, Janet-Maitre M, Castro ÍA, Mackel JJ, Rosen DA, et al. A chronic murine model of pulmonary Acinetobacter baumannii infection enabling the investigation of late virulence factors, long-term antibiotic treatments, and polymicrobial infections [Internet]. bioRxiv; 2024 [cited 2025 Feb 24]. p. 2024.09.17.613469. Available from: https://www.biorxiv.org/content/10.1101/2024.09.17.613469v2
