# Supplementary material for: Acinetobacter phages use distinct strategies to breach the capsule barrier
Source: PLoS Pathog. 2025 Sep 29;21(9):e1013536. doi: 10.1371/journal.ppat.1013536 (PMC12507263; doi:10.1371/journal.ppat.1013536)
Supplement: S10 Table — List of all primers used in the paper. (PDF) [file ppat.1013536.s020.pdf]

Table S10: Primers

List of all primers used in the paper.

Name	Sequence	Description
**F_upstream_wzy_UPAB1**	GATTTTATATTATAACTTTAGCTTTTGTATTTATGGG	Amplification of ~300bp region upstream of *wzy* for generation of deletion mutant in UPAB1
**R_upstream_wzy_UPAB1**	GCCTACACAATCGCTATAAACACCTGATTTAAAAATAACATATTTCAAATTTC	
**F_downstream_wzy_UPAB1**	GAGGATATTCATATGTAGAATATTTTATATTTTTCTCTCAATCTTAGAAGTTC	Amplification of ~300bp region downstream of *wzy* for generation of deletion mutant in UPAB1
**R_downstream_wzy_UPAB1**	CTCACCTTTATATATAAAAGATCCAAATTTTTCC	
**P1**	AGCGATTGTGTAGGCTGGAGCTG	Amplification of the FRT-flanked apramycin resistance cassette from pKD4 for generation of *wzy* mutant in UPAB1
**P2**	CATATGAATATCCTCCTTAGTTCCTATTCCG	
**F_Apr**	CAGGATGAGGATCGTTTCGCTC GGTTCGTAAACTGTAATGCAAGTAGC	Amplification of apramycin cassette from pUC18T-miniTn7T-Apr
**R_Apr**	CTAGAGTAAGCGTTAATAATTCAG CCAATCGACTGGCGAGC	
**F_pJNW684_markerswap**	ATTATTAACGCTTACTCTAGAATGCG	Linearization of pJNW684 to exchange antibiotic resistance maker
**R_pJNW684_markerswap**	GCGAAACGATCCTCATCCTGTCTC	
**F_carO_ACICU**	CATGCATGAGCTCACTAGTGCGAAGATCACATG ACTCGATTGG	Amplification of *carO* from AbACICU for insertion in pUC18T-miniTn7T-LAC-zeo
**R_carO_ACICU**	GCAAGGCCTTCGCGAGGTACCTTACCAGAAGAA GTTCACACC	
**F_carO_19606**	CATGCATGAGCTCACTAGTGGAAGATCACATGA CTCGATTGG	Amplification of *carO* from 19606 for insertion in pUC18T-miniTn7T-LAC-zeo
**R_carO_19606**	GCAAGGCCTTCGCGAGGTACCTTACCAGTAGAA GTTTACACCAAC	
**F_upstream_carO_398**	GTC GAC TCT AGA GGA TCC CCG GGA GAT TTA GAA GAT GAA TTA GGC G	Amplification of regions surrounding *carO* in 398 for insertion into pEX18Ap for generation of *carO* mutants
**R_upstream_carO_398**	GGA GAA AAC GTA AAT AGT AAA AAA ACG AGC TTC G	
**F_downstream_carO_398**	TTA CTA TTT ACG TTT TCT CCT TAA GAA AAG GC	
**R_downstream_carO_398**	CGA ATT CGA GCT CGG TAC CCG GGC TGG TAC AAC AAC TGA GCC	
**F_pEX18**	ATGCCTGCAGGTCGACTCTAGAGG	Linearization of pEX18Ap
**R_pEX18**	GCAAGCTTGGCACTGGCCGT	
**F_upstream_pgrD**	ACG GCC AGT GCC AAG CTT GCT CCT TCG TTA AGC GTA TTC G	Amplification of regions surrounding pgrD in MC47.2 for insertion into pEX18Ap for generation of *pgrD* mutants
**R_upstream_pgrD**	ACA CTA GCA CAC TTG AGC TGT TTG GTA AAG	
**F_downstream_pgrD**	CAG CTC AAG TGT GCT AGT GTC TGA AAA CGG C	
**R_downstream_pgrD**	TAG AGT CGA CCT GCA GGC ATA AGC AAC GAC ATT AGC ACG C	
**F_pEX18_screen**	GGGTAACGCCAGGGTTTTCC	Primers to screen for successful insertion into pEX18Ap plasmid
**R_pEX18_screen**	CCGGCTCGTATGTTGTGTGG	
**F_pUC18T_LAC**	GGG GAT CCA CTA GTG AGC TCG	Linearization of pUC18T-miniTn7T-Apr-LAC
**R_pUC18T_LAC**	AAT TCC TCG AGA AGC TTG GG	

**F_pgrD**	CGA GCT CAC TAG TGG ATC CCC CGG AAC GTA GCA AGG AGA AA	Amplification of *pgrD* from MC47.2 for insertion into pUC18T-miniTN7T-Apr-LAC for complementation, also used to screen *pgrD* mutants for successful deletion after sucrose selection
**R_pgrD**	CCC AAG CTT CTC GAG GAA TTA ACC CCG CAT AAC CCA CTA C	
**F_pgrF**	CGA GCT CAC TAG TGG ATC CCC AAA ACT ACG GGT TGA ACG TC	Amplification of *pgrD* from MC47.2 for insertion into pUC18T-miniTN7T-Apr-LAC for complementation
**R_pgrF**	CCC AAG CTT CTC GAG GAA TTA TCG ACT CGT TTA TGG CAG C	
**F_pUC18T_screen**	TTGACAAAGGGAATCAGG	Primers to screen for successful insertion of genes into pUC18T-miniTn7T plasmids
**R_pUC18T_screen**	ACTTATCTGGTTGGCCTGC	
**F_attTn7_screen**	GTTGGAAGTTGCGGATGTTAC	Primers to screen for successful insertion of genes into the attTn7 site using the pUC18T-miniTn7 plasmids
**R_attTn7_screen**	TTGGCGAAGTCAGTAACTG	
